# Acetyl-CoA acyltransferase 2 palmitoylation drives liver fibrosis by inducing hepatic stellate cell ferroptosis

**DOI:** 10.1016/j.redox.2026.104035

**Published:** 2026-01-17

**Authors:** Jianxiong Han, Zhongkang Yan, Zhiran Sun, Wenyuan Dang, Bao Li, Shuangshuang Li, Xinru Lv, Lin Ni, Anyuan He, Pengying Gu, Feifei Wang, Lili Wang, Xingyuan Yang

**Affiliations:** a School of Life Sciences and Medical Engineering, Institute of Physical Science and Information Technology, Anhui University, Hefei, Anhui, 230601, PR China; b School of Life Sciences and Medical Engineering, Anhui University, Hefei, Anhui, 230601, PR China; c School of Life Sciences, Anhui Medical University, Hefei, Anhui, 230032, PR China; d Department of Geriatrics, The First Affiliated Hospital of USTC, Division of Life Sciences and Medicine, University of Science and Technology of China, Hefei, Anhui, 230001, PR China

**Keywords:** ACAA2, AMPK, Ferroptosis, Liver fibrosis, Palmitoylation, HSC

## Abstract

Hepatic fibrosis is a major driver of mortality in metabolic dysfunction-associated steatotic liver disease (MASLD)—previously known as non-alcoholic fatty liver disease (NAFLD). While hepatic stellate cell (HSC) activation and myofibroblast accumulation are central to fibrogenesis, the regulatory mechanisms remain incompletely understood. Acetyl-CoA acyltransferase 2 (ACAA2), a pivotal enzyme in fatty acid oxidation, has been implicated in lipid metabolism but has not been investigated as a therapeutic target in MASLD. Here, we show that ACAA2 upregulation in HSCs exacerbates hepatic fibrosis by promoting ferroptosis-associated transcriptional programs, whereas ACAA2 inhibition attenuates both ferroptosis and fibrogenesis in preclinical models. Mechanistically, ACAA2 palmitoylation governs its subcellular localization and function, and blocking this modification suppresses HSC activation via AMPK pathway stimulation, thereby mitigating fibrosis. Our study establishes ACAA2 palmitoylation as a druggable node for antifibrotic therapy, offering novel insights into metabolic regulation of hepatic fibrosis.

## Highlights

• Hepatocyte-specific ACAA2 suppression attenuates ferroptosis and liver fibrosis through AMPK-mediated signaling.
• ACAA2 ablation in hepatic stellate cells (HSCs) directly impedes their activation and profibrotic phenotype.
• Palmitoylation-dependent regulation of ACAA2 drives ferroptosis progression and fibrogenesis.
• Inhibition of ACAA2 ameliorates fibrosis in both carbon tetrachloride (CCl_4_) and metabolic dysfunction-associated steatohepatitis (MASH) murine models.

## Graphical abstract

**Figure:**
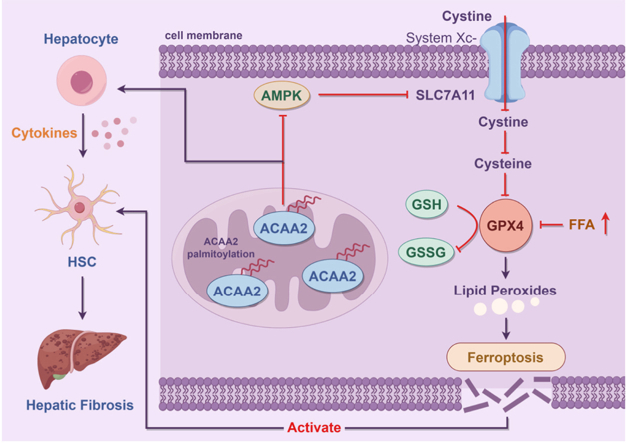

## 1 Introduction

Chronic liver diseases—encompassing metabolic dysfunction-associated steatotic liver disease (MASLD), metabolic associated steatohepatitis (MASH), fibrosis, and hepatocellular carcinoma (HCC)—constitute an escalating global health crisis, accounting for a disproportionate share of mortality and morbidity [[1], [2], [3]]. Although etiologically heterogeneous, these conditions converge on shared pathogenic hallmarks: sustained hepatic stress, dysregulated inflammation, and programmed hepatocyte death [4,5]. Among these, hepatic fibrosis represents a pivotal juncture in MASLD progression, its rising prevalence paralleling epidemics of metabolic syndrome, obesity, and type 2 diabetes mellitus (T2DM). Driven by maladaptive extracellular matrix (ECM) deposition in response to chronic injury, fibrosis arises from diverse insults, including viral hepatitis, MASH, and diabetic hepatopathy [[6], [7], [8]]. Notably, cirrhosis—the end-stage of fibrotic remodeling—ranks as the 11th leading cause of death globally, underscoring the urgency of elucidating its molecular underpinnings. Despite advances, the mechanistic links between metabolic dysregulation, hepatocyte death pathways, and fibrogenesis remain elusive, hampering therapeutic development.

Emerging evidence implicates ferroptosis, an iron-catalyzed form of regulated cell death (RCD), as a critical mediator of hepatic pathophysiology [[9], [10], [11]]. Distinct from apoptosis or pyroptosis, ferroptosis is mechanistically defined by lethal lipid peroxidation, governed primarily by the SLC7A11/GPX4 axis and modulated by ACSL4, FSP1, and AMPK [[12], [13], [14]]. Iron overload exacerbates this process by amplifying reactive oxygen species(ROS), thereby inactivating GPX4 and precipitating cell death [15,16]. Intriguingly, ferroptosis has been mechanistically linked to metabolic disorders, including MASLD and HCC, yet its role in HSC activation and fibrogenesis remains unexplored.

Central to this metabolic-cell death interplay is AMP-activated protein kinase (AMPK), a master regulator of energy homeostasis [[17], [18], [19]]. AMPK's heterotrimeric structure, featuring α, β, and γ subunits, enables its activation via α-subunit phosphorylation (Thr172) during energy stress [20]. Beyond its canonical metabolic roles, AMPK modulates ferroptosis through SLC7A11 suppression, though its functional impact on fibrotic progression is unknown.

A potential nexus between metabolism and ferroptosis lies in mitochondrial β-oxidation, exemplified by acetyl-CoA acyltransferase 2 (ACAA2). This thiolase catalyzes the terminal step of fatty acid degradation, generating acetyl-CoA while contributing to ketogenesis [21]. Although ACAA2 has been implicated in adipogenesis and heavy metal toxicity [22,23], its hepatic functions—particularly in fibrotic contexts—are undefined. Prior work suggests ACAA2 may suppress lipogenesis in mammary tissue, but its regulation by post-translational modifications (PTMs) and role in liver disease remain uncharted.

Protein palmitoylation, the reversible thioesterification of cysteine residues by palmitate, emerges as a critical PTM modulating protein trafficking, stability, and activity [24,25]. Whether ACAA2 undergoes functional palmitoylation to influence hepatic metabolism or ferroptosis is unestablished. Here, we identify ACAA2 palmitoylation as a novel mechanistic link between ferroptotic hepatocyte death and HSC-driven fibrogenesis, bridging metabolic dysregulation with progressive liver injury.

## 2 Materials and methods

### 2.1 Human samples

The human samples and data were collected from the First Affiliated Hospital of USTC, Division of Life Sciences and Medicine. Participants with liver fibrosis who were willing to participate in the study were included. The patients did not have the following exclusion criteria [1]: use of corticosteroids within the last 3 months [2]; acute infection [3]; autoimmune disease [4]; the severe cardiovascular and cerebrovascular diseases. The human liver tissue was collected during the surgery which was cut out for pathological analysis. The study was conducted with the approval of the ethics committees of the First Affiliated Hospital of USTC, Division of Life Sciences and Medicine (No.2022-086). Written informed consent was provided by all participants.

### 2.2 Animal studies

All animal experiments were conducted in accordance with the ARRIVE guidelines and were approved by the Institutional Animal Care and Use Committee of Anhui University [Protocol No. IACUC(AHU)-2023-065]. Mice were maintained under specific pathogen-free conditions at 22 ± 1 °C with 50 ± 10 % humidity, a 12-h light/dark cycle (lights on at 07:00), and ad libitum access to food and water.

### 2.3 Animal models and experimental design

#### 2.3.1 Diet-induced obesity model

Ten-week-old male C57BL/6J mice (n = 7; Cyagen Biosciences, Suzhou, China) were randomly assigned to either High-fat diet (HFD; 60 % kcal from fat, 20 % protein, 20 % carbohydrate; D12492, Research Diets), Normal chow diet (NCD; 10 % kcal from fat; D12450J, Research Diets) for 12 weeks (n = 7/group).

### 2.4 Methionine-choline deficient (MCD) model

Age-matched C57BL/6J mice (n = 7/group) were fed either Standard chow or MCD diet (A02082002BR, Guangdong Medical Lab Animal Center) for 6 weeks.

### 2.5 Chemical-induced fibrosis model

Ten-week-old male C57BL/6J mice received intraperitoneal injections of 20 % CCl4 (v/v in olive oil; 2 μl/g body weight; Sigma-Aldrich) twice weekly Vehicle control (olive oil alone) for 8 weeks (n = 7/group).

### 2.6 Genetic modification studies

*ACAA2* knockout mice (*ACAA2*-KO; C57BL/6J background) and wild-type littermates were generated by Cyagen Biosciences. Ten-week-old male mice received AAV8-control (1 × 10^11^ vg/mouse; HB-AAV-001, Hanbio) AAV8-*ACAA2* (1 × 10^11^ vg/mouse; HB-AAV-002, Hanbio) via tail vein injection, followed by HFD feeding for 8 weeks (n = 7/group).

### 2.7 Sample collection and processing

After a 12-h fasting period (water ad libitum), mice were anesthetized with pentobarbital (50 mg/kg, i.p.). Blood was collected via abdominal aorta puncture and centrifuged (3,000g, 15 min, 4 °C) to obtain serum for biochemical analysis (AU5800, Beckman Coulter). Livers were fixed in 4 % paraformaldehyde (24–48 h) for histology. Snap-frozen in liquid nitrogen and stored at −80 °C for molecular analyses.

### 2.8 Statistical analysis

Sample sizes were determined based on power calculations (α = 0.05, power = 0.8) using preliminary data. Randomization was performed using GraphPad Prism v9.0. Researchers were blinded to group allocation during data collection and analysis.

### 2.9 Histological and Sirius Red staining analysis

Fixed tissues were dehydrated, embedded in paraffin, and sectioned into 5 μm-thick slices. Sections were then stained with hematoxylin for 5–10 min at room temperature to visualize nuclei, followed by rinsing in running tap water and differentiation in 1 % acid alcohol if necessary. After bluing in 0.2 % ammonia water or saturated lithium carbonate solution, sections were counterstained with eosin for 1–3 min to highlight cytoplasm and extracellular matrix components. Stained sections were dehydrated through ascending ethanol series, cleared in xylene, and mounted with a resinous medium. Tissue morphology was evaluated under a bright-field microscope, and representative images were captured for further histological analysis.

Paraffin-embedded liver tissue sections (4–5 μm) were deparaffinized in xylene and rehydrated through a graded ethanol series to distilled water. Sections were then incubated with 0.1 % Sirius Red solution in saturated picric acid for 60 min at room temperature to specifically stain collagen fibers. Following staining, sections were rinsed briefly in 0.5 % acetic acid to remove unbound dye, dehydrated through graded ethanol series, cleared in xylene, and mounted with a resinous mounting medium. Stained sections were imaged using a bright-field microscope, and the extent of collagen deposition was quantified by measuring the Sirius Red-positive area using ImageJ software or other image analysis tools, with results expressed as the percentage of positively stained area relative to the total tissue area.

Paraffin-embedded liver tissue sections (4–5 μm) were deparaffinized in xylene and rehydrated through a graded ethanol series, followed by antigen retrieval in citrate buffer (pH 6.0) at 95 °C for 20 min. Endogenous peroxidase activity was quenched by incubation with 3 % hydrogen peroxide for 10 min at room temperature, and non-specific binding was blocked using 5 % bovine serum albumin in phosphate-buffered saline (PBS) for 30 min. Sections were then incubated overnight at 4 °C with primary antibodies against [target protein] at optimized dilutions, followed by incubation with biotin-conjugated secondary antibodies for 30 min at room temperature. Signal detection was performed using a streptavidin–horseradish peroxidase complex and DAB, and nuclei were counterstained with hematoxylin. Stained sections were dehydrated, cleared, and mounted with a coverslip, and images were captured using a bright-field microscope.

Quantitative analysis of IHC staining (IHC scores) and Sirius red-positive areas was conducted using Image J software (NIH).

### 2.10 Cell culture

HepG2 and LX2 cells were maintained in high-glucose Dulbecco's Modified Eagle Medium (DMEM; Biological Industries, Israel) supplemented with 10 % fetal bovine serum (FBS; Gibco), 100 U/mL penicillin, and 100 μg/mL streptomycin (Thermo Fisher Scientific). AML12 cells were cultured in DMEM/F-12 (1:1) medium (Viva Cell Biosciences). All cells were incubated at 37 °C in a humidified 5 % CO_2_ atmosphere.

### 2.11 Co-culture of hepatocytes and HSCs

A Transwell co-culture system (Corning Costar) was employed to assess intercellular communication. LX2 cells were seeded in 6-well plates and allowed to adhere for 24 h. Subsequently, HepG2 cells were plated onto Transwell inserts (0.4 μm pore size; Corning) and placed above the LX2 monolayer. This setup permits bidirectional exchange of soluble factors while preventing direct cell contact. After 48 h of co-culture, cells were harvested for downstream analysis.

### 2.12 Plasmid expression and siRNA-Mediated knockdown

The ***ACAA2*** gene was amplified from a C57BL/6 mouse liver cDNA library and cloned into pcDNA3.0-HA vectors. Transfection was performed using Lipo8000 (Beyotime), with plasmids combined in Opti-MEM Medium (Gibco) to form plasmid-lipid complexes before addition to cells and incubation for 24 h.

For *ACAA2* knockdown in HepG2 and LX2 cells, siRNA oligonucleotides (General Biol) were used with the following sequences: Sense:5′-GAGAAGAAUGUGACAAAUATT-3′ Antisense: 5′-UAUUUGUCACAUUCUUCUCTT-3′

A total of 100 pmol of siRNA was transfected into cells plated on 35-mm dishes using Lipo8000. After 48 h, cells were harvested for downstream assays.

### 2.13 Primary mouse cell isolation and culture

Mouse livers were perfused in situ via the portal vein with pre-warmed, calcium- and magnesium-free Hanks’ Balanced Salt Solution to efficiently remove residual blood, followed by perfusion with a collagenase-containing buffer to enzymatically digest the hepatic tissue. Excised livers were then carefully minced into small fragments and subjected to additional ex vivo digestion under sterile conditions to achieve complete tissue dissociation. The resulting cell suspension was filtered through a 70-μm nylon cell strainer to eliminate undigested tissue fragments and large debris. Parenchymal hepatocytes were enriched by low-speed centrifugation, whereas the remaining non-parenchymal cell fraction was collected and subjected to discontinuous density gradient centrifugation to isolate HSCs based on their characteristic buoyant density. Isolated hepatocytes and HSCs were resuspended in cell type-specific culture media, seeded onto collagen-coated or uncoated culture vessels as appropriate, and maintained under standard culture conditions at 37 °C in a humidified atmosphere containing 5 % CO_2_. Cell viability and purity were assessed by trypan blue exclusion and confirmed by morphological evaluation and expression of cell-specific markers, including albumin for hepatocytes and desmin or glial fibrillary acidic protein (GFAP) for HSCs, ensuring the reliability and reproducibility of downstream in vitro assays.

### 2.14 Quantitative RT-PCR analysis

Total RNA was extracted from cells or tissue samples using Trizol and RNA quality and concentration were assessed by spectrophotometry. Complementary DNA (cDNA) was synthesized from total RNA using Genestar reverse transcriptase according to the manufacturer's instructions. For quantitative real-time PCR (qRT-PCR), 50 ng of cDNA was amplified with 0.25 μM of gene-specific primers targeting *ACTA2, CXCL10, CXCL1, IL8, COL1A1*, and *TGF-β1*, or primers for *GAPDH* as an internal control. Reactions were performed on an ABI Real-Time PCR Detection System using a standard SYBR Green-based protocol, and each sample was run in triplicate. Relative mRNA expression levels were calculated using the 2^−ΔΔCt method, with target gene expression normalized to GAPDH, and data were expressed as fold change relative to the corresponding control.

### 2.15 Western blotting

Cells or tissue samples were lysed in RIPA buffer supplemented with protease and phosphatase inhibitors, and protein concentrations were determined using a BCA assay. Equal amounts of protein (20–40 μg) were denatured by boiling in Loading buffer and separated by SDS-PAGE on 8–12 % polyacrylamide gels, followed by transfer onto nitrocellulose membranes (Millipore). Membranes were blocked with 5 % non-fat milk or bovine serum albumin in Tris-buffered saline containing 0.1 % Tween-20 (TBST) for 1 h at room temperature and incubated overnight at 4 °C with primary antibodies against the target proteins at optimized dilutions. After washing with TBST, membranes were incubated with horseradish peroxidase-conjugated secondary antibodies for 1 h at room temperature. Protein bands were visualized using an enhanced chemiluminescence (ECL) detection system and imaged with a chemiluminescence imaging system (Tanon 5200). Quantification of band intensity was performed using ImageJ software or equivalent, and protein expression levels were normalized to appropriate loading controls, such as β-tubulin or GAPDH.

### 2.16 Triglyceride measurement

Cells were seeded into 35 mm plates and incubated with OA/PA for 1–8 h. Next, the cells were collected and lysed ultrasonically in PBS buffer and the TGs were measured using a TG assay kit. Specifically, 2.5 μL of cell lysate was mixed with 250 μL of working solution (containing 100 mmol/L Tris-HCl, 3000 U/L lipase, 0.5 mmol/L ATP, 1000 U/L glycerol kinase, 5000 U/L glycerol-3-phosphate oxidase, 1000 U/L peroxidase, 1.4 mmol/L 4-aminoantipyrine, and 3 mmol/L *p*-chlorophenol). The mixture was added to a microplate and incubated at 37 °C for 10 min, after which absorbance was measured at 500 nm using a microplate reader.Total cellular protein content was also quantified using the BCA Protein Assay kit. Intracellular TG concentration were normalized to total cellular protein concentration and expressed as mmol/mg protein.

### 2.17 Lipid droplet staining

Cells were washed with phosphate-buffered saline (PBS) and fixed in 4 % paraformaldehyde for 15–20 min at room temperature to preserve cellular morphology. Following fixation, cells were rinsed three times with PBS and incubated with 1 μg/mL BODIPY 493/503 to specifically label neutral lipid droplets for 15–30 min in the dark. Nuclei were counterstained with 1 μg/mL DAPI for 5 min, followed by additional PBS washes to remove unbound dye. Stained cells were mounted using an anti-fade mounting medium and imaged using a Leica fluorescence microscope equipped with a 63 × oil immersion objective. Fluorescence intensity and lipid droplet number or area were quantified using ImageJ software or comparable image analysis tools to assess intracellular lipid accumulation.

### 2.18 Analysis of the mitochondrial membrane potential

LX2 cells (2 × 10^5^/ml) were plated in 6-well plates treated with various concentrations of Lip-1. After 24 h, added with 1 mL Rhodamine 123 (2 μmol/L) staining working solution, incubated at 37 °C for 30 min in a cell incubator. Subsequently, the cells were rinsed twice with fresh pre‐warmed complete medium at 37 °C and observed under a Confocal Laser Scanning Microscope (63 × ,Leica) at an excitation wavelength of 507 nm and an emission wavelength of 529 nm.

### 2.19 Cell viability assay

Cell viability was evaluated using a MTT kit (Beyotime) per the manufacturer's protocol. Briefly, LX2 cells were seeded in 96-well plates at 5000 cells per well. Cells were incubated with fresh media containing MTT solution for 4 h prior to addition of dimethyl sulfoxide (DMSO) to dissolve formazan. Samples were assessed using a microplate reader, and absorbance was read at 490 nm.

### 2.20 TGF-β1 ELISA

Latent TGF-β1 in liver tissue homogenates was activated by adding 20 μL of 1 mol/L HCl to 40 μL of homogenate, followed by incubation at room temperature for 10 min. Samples were neutralized with 20 μL of 1.2 mol/L NaOH/0.5 mol/L HEPES and diluted 60-fold prior to analysis. TGF-β1 levels were measured using an ELISA assay. Briefly, plates were coated with mouse TGF-β1 capture antibodies and incubated overnight at room temperature. After washing with PBST, plates were blocked with 5 % PBST for 1 h and incubated with activated samples and standards for 2 h. Plates were then incubated with a mouse TGF-β1 detection antibody for 2 h, followed by streptavidin–HRP for 20 min. Color was developed using TMB substrate for 20 min and stopped with 2 mol/L H_2_SO_4_. Absorbance was measured at 450 nm using a microplate reader.

### 2.21 Ferrous ion, glutathione and malondialdehyde measurement

Ferrous ion (Fe^2+^) content in cells or tissue samples was determined using a commercial assay kit (Solarbio) in accordance with the manufacturer's instructions. Briefly, samples were homogenized and prepared as directed, and the Fe^2+^ concentration was quantified based on the formation of a colored complex, with absorbance measured at the specified wavelength using a microplate reader. Levels of malondialdehyde (MDA) and reduced and oxidized glutathione (GSH and GSSG) were measured using a commercial MDA and GSH/GSSG assay kit (Beyotime) following the manufacturer's protocols. Samples were treated with the appropriate reaction reagents, incubated at the recommended conditions, and the resulting colorimetric or fluorescent signals were detected using a microplate reader. The concentrations of Fe^2+^, MDA, GSH, and GSSG were calculated based on standard curves, and the GSH/GSSG ratio was determined to evaluate cellular redox status and lipid peroxidation levels.

### 2.22 Detection of ACAA2 palmitoylation

ACAA2-MYC was immunoprecipitated using anti-MYC antibody and Protein A/G-Sepharose beads (Beyotime, P2055). Samples were treated with hydroxylamine (HAM) or control buffer, labeled with biotin-BMCC, and analyzed by SDS-PAGE/Western blotting. Membranes were blocked in 1 % BSA/TBST, probed with HRP-streptavidin, and detected with anti-ACAA2 and HRP-conjugated secondary antibodies.

### 2.23 Nuclear and cytoplasmic fractionation

Nuclear and cytoplasmic fractions were isolated using a commercial nuclear and cytoplasmic extraction kit or a standard differential centrifugation protocol according to the manufacturer's instructions. Briefly, cells were harvested, washed with ice-cold phosphate-buffered saline, and resuspended in cytoplasmic extraction buffer supplemented with protease and phosphatase inhibitors. After incubation on ice with intermittent vortexing, samples were centrifuged at high speed to separate the cytoplasmic supernatant from the nuclear pellet. The nuclear pellet was subsequently washed and lysed in nuclear extraction buffer with vigorous vortexing to ensure complete nuclear protein extraction. Both fractions were clarified by centrifugation, and protein concentrations were determined using a BCA assay. The purity of nuclear and cytoplasmic fractions was validated by immunoblotting for compartment-specific markers, such as lamin B1 for the nucleus and GAPDH for the cytoplasm.

### 2.24 Separation of soluble and vesicular factors by EV-depleted conditioned medium preparation

Conditioned medium (CM) was collected from donor cells cultured in medium containing 5 % FBS for 24 h. CM was cleared by sequential centrifugation at 500×*g* for 10 min, 2000×*g* for 20 min and 10,000×*g* for 20 min at 4 °C, and then passed through a 0.22 μm filter. For EV isolation, cleared CM was ultracentrifuged at 100,000×*g* for 90 min at 4 °C. The pellet was washed in PBS and centrifuged again at 100,000×*g* for 60 min.

### 2.25 Statistical analysis

Data are presented as **mean ± SEM**. Significance was assessed via **Student's t-test** or one-way ANOVA with Tukey's multiple comparisons test (∗***p < 0.05***, ∗∗p < 0.01, ∗∗∗p < 0.001) using **GraphPad Prism 8**.

### 2.26 Data availability

The **ACAA2** sequence is available in GenBank (MGI:1098623). All supporting data are included in the manuscript or supplementary materials.

## 3 Results

### 3.1 Elevated hepatocytic ACAA2 expression correlates with hepatic fibrogenesis

**Fig. 1:**
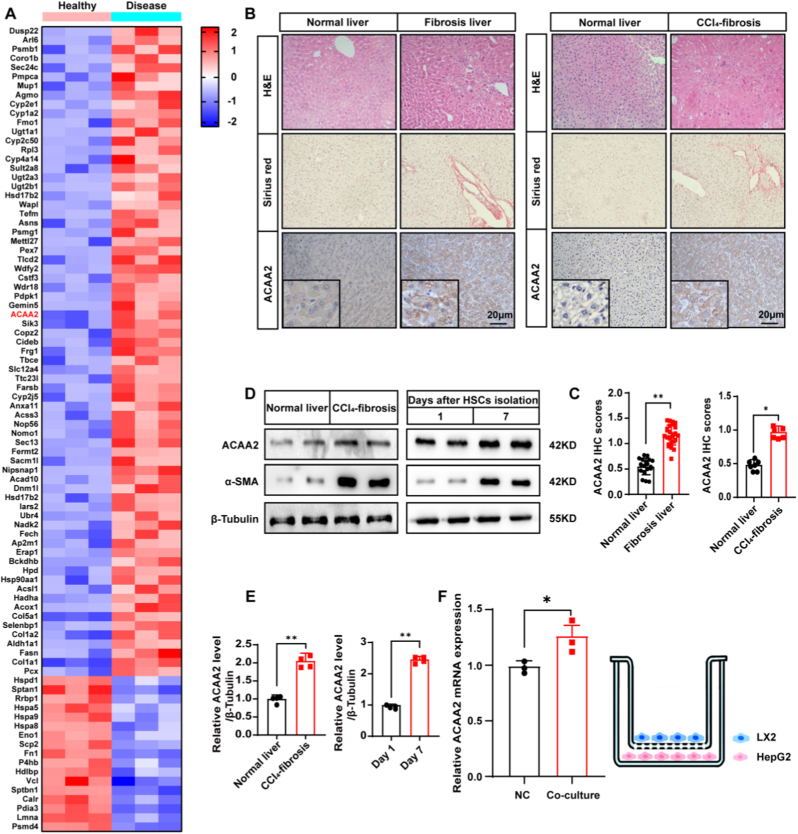
Up-regulated ACAA2 expression in HSCs and hepatocytes is associated with liver fibrosis **(A)** Heatmap displaying relative expression of genes in the liver (n = 3); **(B)** H&E staining, ACAA2 immunohistochemistry (IHC), and Sirius red staining were performed in normal (n = 18), human liver fibrotic/cirrhotic tissues (n = 25), and normal (n = 7), CCl_4_-induced mouse fibrosis tissues (n = 6). Representative images of H&E staining, ACAA2, and Sirius red staining are shown in the panel. **(C)** Quantitative analysis of ACAA2 staining. **(D-E)** Immunoblotting confirmation of the related protein in the liver and primary mouse HSCs. **(F)** HepG2 cells increases ACAA2 mRNA expression levels in LX2 cells in the co-culture model. The right schematic represents the Transwell co-culture model of HSCs and HepG2 cells (n = 3). Data are shown as mean ± SEM. ∗*p* < 0.05, ∗∗*p < *0.01, ∗∗∗*p* < 0.001, NS = not significant. *t*-test.

To elucidate molecular mechanisms underlying fibrotic liver injury, we performed comparative analyses of healthy and CCl_4_-induced fibrotic murine livers. Unsupervised clustering revealed significant upregulation of ACAA2 in fibrotic specimens (Fig. 1A). Histopathological quantification demonstrated a 2.3-fold increase in Sirius red-positive fibrotic areas concomitant with elevated ACAA2 immunoreactivity in human fibrotic livers versus healthy controls. This phenotype was recapitulated in our murine CCl_4_ model, showing both increased collagen deposition (1.8-fold, p < 0.01) and ACAA2 protein abundance (Fig. 1B–C). Western blot analysis confirmed this differential expression, with fibrotic tissues exhibiting 1.9 ± 0.4-fold higher ACAA2 levels.We analyzed the publicly available National Cancer for Biotechnology Information Gene Expression Omnibus (GEO) database (Fig. S1). Quantitative analysis (GSE158723) showed that ACAA2 levels were increased in the activated group compared with the quiet group (mean log_2_-ratio = +0.54, corresponding to ∼1.45-fold upregulation).

Investigation of hepatic stellate cell (HSC) activation dynamics revealed temporal regulation of ACAA2. Primary murine HSCs isolated during progressive activation (days 1–7 post-isolation) showed concomitant upregulation of ACAA2 and α-smooth muscle actin (α-SMA), with maximal expression at day 7 (2.7-fold and 3.2-fold increases respectively; Fig. 1D–E). Furthermore, heterotypic cellular interactions enhanced *ACAA2* transcription, as evidenced by 1.9-fold greater mRNA levels in HepG2-co-cultured HSCs versus monocultures (Fig. 1F).

### 3.2 ACAA2 promotes hepatic stellate cell activation and ferroptosis through AMPK pathway modulation

**Fig. 2:**
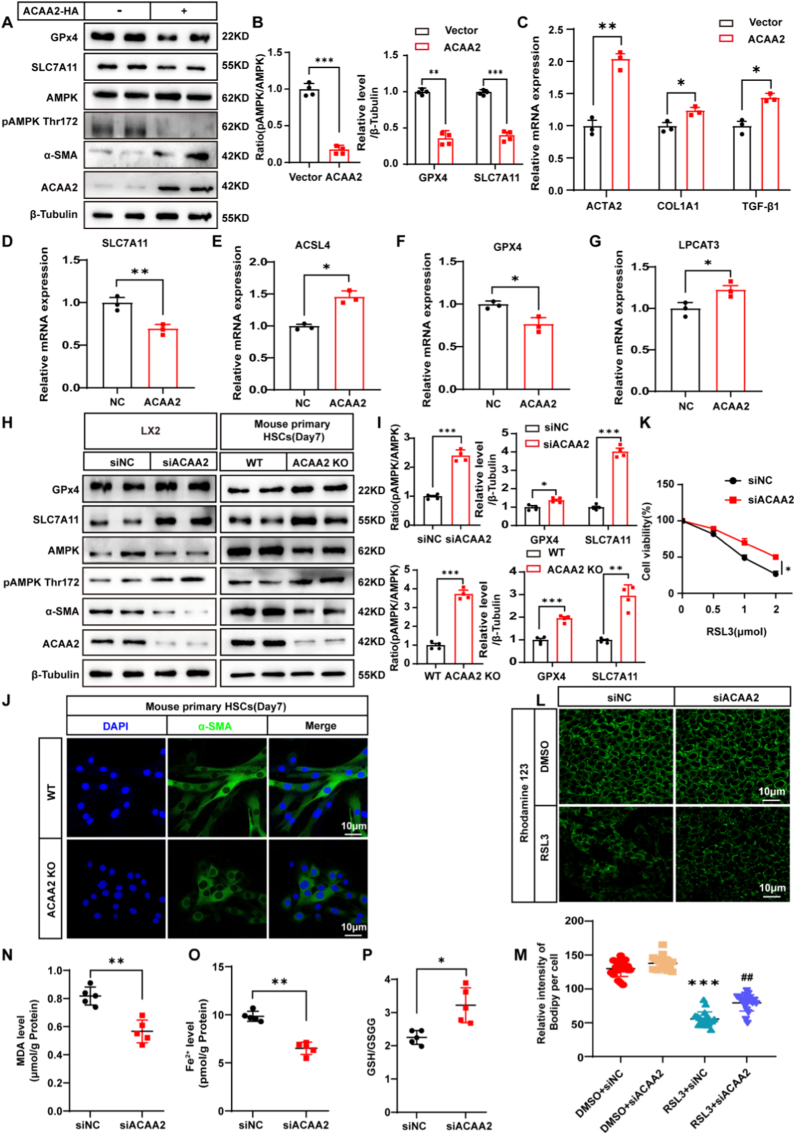
Inhibition of ACAA2 stimulates SLC7A11/GPX4 pathway and decreases liver fibrosis **(A-B)** SLC7A11/GPX4 pathway activity is increased by overexpression of ACAA2 in LX2 cells. **(C-G)** Overexpression of ACAA2 increased mRNA expression of ferroptosis and HSC activation related genes compared to vector (n = 3). **(H)** Left panel shows ACAA2 siRNA facilitates SLC7A11/GPX4 pathway in LX2 cells. The right panel shows ACAA2 KO mice primary HSCs increased SLC7A11/GPX4 pathway compared to WT mice. **(I)** Band intensity was quantified by densitometry. **(J)** Representative double immunofluorescence images of α-SMA (green) and DAPI (blue) in the primary HSCs isolated from normal or ACAA2-KO mouse livers and cultured for seven days. **(K)** LX2 cells were transfected with nontargeting RNA (siNC) or small interfering RNA targeting either ACAA2 (siACAA2). Viability of cells treated with indicated doses of RSL3. **(L-M)** Mitochondrial membrane potential measured by Rhodamine123 (green) in LX2 cells of four groups. **(N)** Level of MDA (n = 5). **(O)** Level of Fe^2+^ (n = 5). **(P)** Ratio of GSH to GSSG (n = 5). Data presented are means ± SD, *t*-test. The differences among four groups were statistically analyzed by one-way ANOVA with Tukey's multiple comparisons test.∗*p* < 0.05, ∗∗*p < *0.01, ∗∗∗*p* < 0.001 vs DMSO + siNC; ^#^*p* < 0.05, ^##^*p* < 0.01 vs RSL3+siNC. NS = no significance.

To investigate ACAA2's role in ferroptosis, we analyzed key ferroptotic markers in LX2 cells. Overexpression of ACAA2 significantly suppressed AMPK phosphorylation and downregulated the level of GPX4 and SLC7A11, while concurrently elevating lipid peroxidation-related genes (ACSL4*,* LPCAT3) (Fig. 2A–B, D–G). This was accompanied by increased expression of HSC activation (ACTA2*, TGF-β1*) and fibrogenic (COL1A1) markers (Fig. 2A–C), suggesting a dual role for ACAA2 in metabolic reprogramming and fibrogenesis.

### 3.3 ACAA2 drives FFAs uptake and sensitizes cells to ferroptotic stress

Given the link between free fatty acid (FFA) metabolism and ferroptosis susceptibility, we assessed lipid dynamics in AML12 hepatocytes. Transient ACAA2-HA overexpression, combined with ATGL inhibition (atglistatin, 20 μM), resulted in progressive lipid droplet (LD) accumulation (>2-fold vs. DMSO controls) following oleic/palmitic acid (OA/PA; 350/150 μM) challenge (Fig. S2A–B). Congruently, ACAA2-overexpressing cells exhibited elevated triglyceride (TG) levels in both hepatocytes and HSCs under lipolytic blockade (Fig. S2E). Genetic ATGL knockdown in LX2 cells recapitulated these phenotypes (Fig. S2C–D), confirming ACAA2's role in lipid metabolism.

### 3.4 ACAA2 depletion attenuates ferroptosis via AMPK activation

In an in vitro MASLD model (OA/PA-treated LX2 cells), ACAA2 knockdown (siACAA2) activated AMPK signaling, restoring SLC7A11/GPX4 expression and reducing ferroptotic markers (MDA, Fe^2+^, GSH/GSSG ratio; Fig. 2H–I, N–P). Crucially, AMPK inhibition (Compound C) reversed the anti-ferroptotic and anti-fibrotic effects of ACAA2 deletion (Fig. S4A–B) and activation of AMPK (AICAR) rescues the fibrogenic/ferroptotic phenotype induced by ACAA2 overexpression (Fig. S5A–B), establishing AMPK as the mechanistic node.Quiescent HSCs isolated from ACAA2 KO mice exhibited sustained AMPK phosphorylation, higher SLC7A11/GPX4 levels, and suppressed activation markers (ACTA2*,* COL1A1) during culture-induced activation (7 days; Fig. 2H–J, S3A–B). To directly probe ferroptosis sensitivity, we treated cells with the GPX4 inhibitor RSL3. ACAA2 deficiency augmented mitochondrial membrane potential (green fluorescence probe) and conferred resistance to RSL3-induced cell death (Fig. 2K–M), underscoring its gatekeeper role in ferroptotic commitment.

### 3.5 ACAA2-mediated hepatocyte-HSC crosstalk drives fibrogenic activation

To elucidate the role of hepatocyte-specific ACAA2 upregulation in liver fibrogenesis and intercellular crosstalk, we established a transwell co-culture system that recapitulates physiologically relevant cell-type-specific interactions while maintaining compartmentalization [26].

**Fig. 3:**
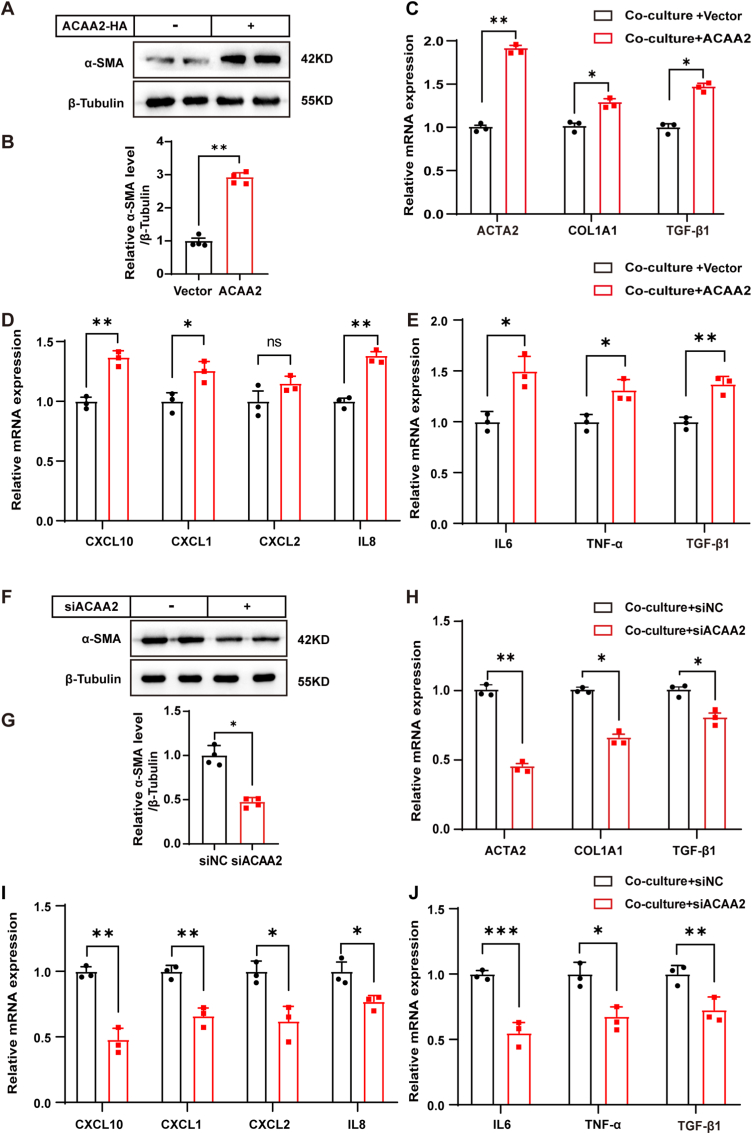
Up-regulation of ACAA2 in hepatocytes promotes HSC activation in the co-culture system **(A-B)** Immunoblotting of LX2 cells co-culture with HepG2 cells. **(C)** Overexpressing ACAA2 enhances mRNA expression of related gene and by RT-PCR (n = 3). **(D-E)** ACAA2 enhances the mRNA level of inflammation related genes and cytokines in HepG2 cells(n = 3). **(F-G)** Knockdown of ACAA2 inhibits HSC activation (n = 3). **(H)** HepG2 cells were treated with ACAA2 siRNA and co-culture with LX2 cells. siACAA2 inhibited co-culture induced up-regulation of mRNA expression of related genes which identified by RT-PCR (n = 3). **(I-J)** ACAA2 inhibits the mRNA level of inflammation related genes and cytokines in HepG2 cells (n = 3).Data presented are means ± SD. NS = not significant. ∗*p* < 0.05; ∗∗*p* < 0.01.∗∗∗*p* < 0.001, *t*-test.

Notably, ACAA2 overexpression in HepG2 hepatocytes potentiated LX2 stellate cell activation, as evidenced by significant induction of canonical activation markers (*α-SMA*, *COL1A1*, *TIMP1*; Fig. 3A–C). Concomitantly, ACAA2-overexpressing hepatocytes exhibited marked upregulation of both profibrotic cytokines (*TGF-β1*, *IL-6*, TNF-α) and chemotactic mediators (*CXCL10*, *IL-8*, *CXCL1*; Fig. 3D and E), suggesting a paracrine signaling axis. To distinguish between soluble and vesicular factors, we collected conditioned medium (CM) from HepG2 cells and subjected it to ultracentrifugation at 100,000×*g* for 90min to remove extracellular vesicles, thereby obtaining EV-depleted conditioned medium. Treatment of LX2 cells with this EV-depleted CM reproduced the fibrogenic effects, indicating that the paracrine mediators are mainly present in the soluble fraction rather than in extracellular vesicles (Fig. S6).

Conversely, siRNA-mediated ACAA2 knockdown in HepG2 cells abrogated the co-culture-induced activation phenotype in LX2 cells (Fig. 2F–H). This intervention also attenuated the hepatocyte inflammatory signature, with pronounced reductions in both inflammatory cytokines and chemokine secretion (Fig. 2I–L).

These data establish ACAA2 as a critical regulator of hepatocyte-to-HSC communication, wherein ACAA2-dependent hepatocyte cytokine production drives stellate cell activation through paracrine mechanisms.

### 3.6 Inhibition of ACAA2 palmitoylation attenuates HSC activation by suppressing ferroptosis

**Fig. 4:**
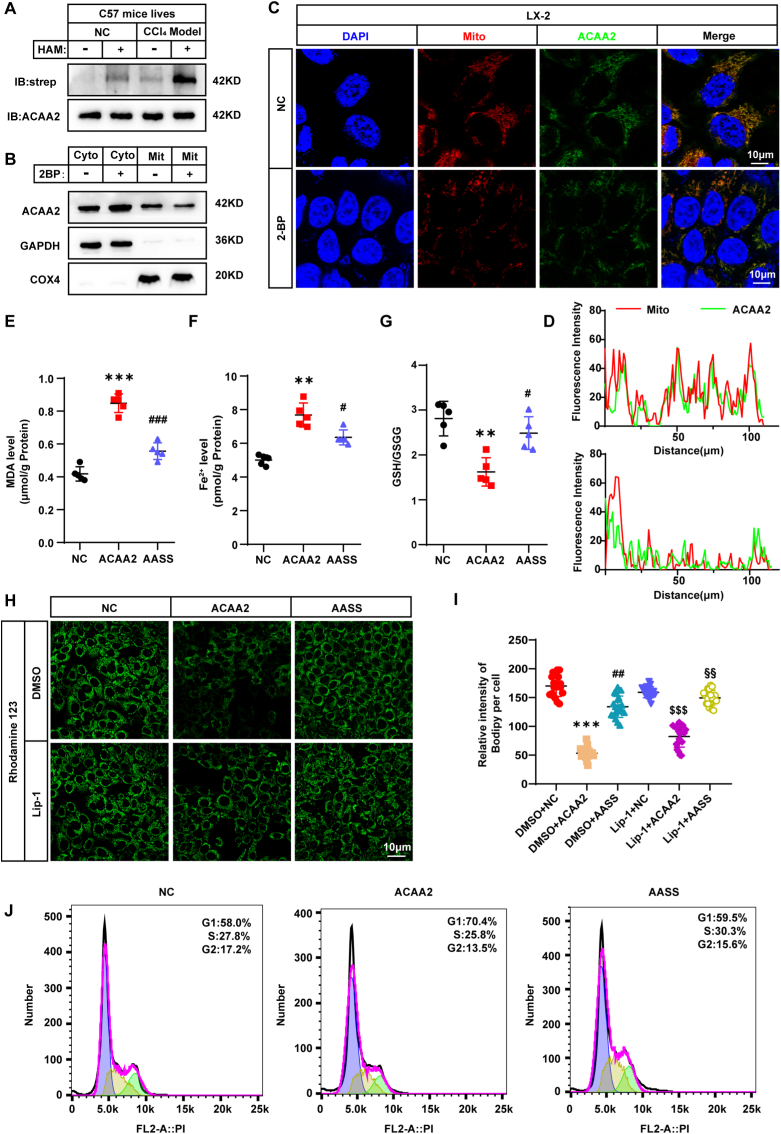
Inhibition of ACAA2 palmitoylation reduces HSC activation by suppression of ferroptosis **(A)** Protein levels of palmitoylated ACAA2 in mice livers. **(B)** The cytosol and mitochondrion fractions were isolated from the 2BP-treated cells and ACAA2 expression in these fractions was analyzed by Western blot. **(C-D)**Analysis using confocal microscopy demonstrates that localization of ACAA2 in LX2 cells treated with 2BP. **(E)** Level of MDA (n = 5). **(F)** Level of Fe^2+^ (n = 5). **(G)** Ratio of GSH to GSSG (n = 5). **(H-I)** The fluorescence image of LX2 cells stained by Rhodamine123. **(J)** Cell cycle distribution was measured by flow cytometry in control, LX2-ACAA2 and LX2-AASS cells (n = 3). Data presented are means ± SD. The differences among multiple groups were statistically analyzed by one-way ANOVA with Tukey's multiple comparisons test.∗*p* < 0.05, ∗∗*p < *0.01, ∗∗∗*p* < 0.001 vs NC; ^#^*p* < 0.05, ^##^*p* < 0.01,^###^*p* < 0.001 vs ACAA2; ^$^*p* < 0.05, ^$$^*p < *0.01, ^$$$^*p* < 0.001 vs Lip-1+NC; ^§§^*p < *0.01 vs Lip-1+ACAA2. NS = no significance.

Using the IP-ABE assay, we observed significantly elevated hepatic ACAA2 palmitoylation in CCl_4_-induced fibrotic mice compared to controls (Fig. 4A). To further verify the results, we sought to identify the palmitoylating enzyme of ACAA2. We analyzed the protein structure and found a region in DHHC21 that interacts with ACAA2, and further validated this result through immunostaining, Knockdown of DHHC21 markedly reduced palmitoylation of ACAA2(Fig. S7).To delineate the functional consequences of palmitoylation, we treated LX2 cells with the palmitoylation inhibitor 2-bromopalmitate (2BP) and performed subcellular fractionation. 2BP treatment markedly reduced mitochondrial ACAA2 localization while enhancing its cytoplasmic accumulation (Fig. 4B–D). Notably, 2BP suppressed lipid droplet (LD) accumulation independently of ACAA2 overexpression (Fig. S8A), suggesting a broader role for palmitoylation in LD regulation.

To dissect the specific contribution of ACAA2 palmitoylation in MASLD pathogenesis, we generated LX2 cells expressing either wild-type ACAA2 (wt-ACAA2) or a non-palmitoylatable mutant (AASS; Table S2). Intriguingly, AASS-expressing cells exhibited elevated pAMPK, GPX4, and SLC7A11 levels alongside reduced α-SMA expression (Fig. S9), coupled with diminished LD formation (Fig. S8B) and inhibited the nuclear translocation of NRF2 and ATF4, thereby reducing their binding to the SLC7A11 promoter(Fig. S10)., implicating palmitoylation in HSC activation and lipid metabolism.

We further interrogated the link between ACAA2 palmitoylation and ferroptosis. While wt-ACAA2 overexpression suppressed LX2 cell proliferation—a phenotype rescued by the ferroptosis inhibitor Lip-1—AASS expression had no such effect (Fig. S11). Mechanistically, wt-ACAA2, but not AASS, triggered ferroptotic hallmarks, including lipid peroxidation (Fig. 4E), redox-active iron accumulation (Fig. 4F), GSH depletion (Fig. 4G), and mitochondrial membrane potential collapse which were abrogated by Lip-1(Fig. 4H and I). Cell cycle analysis revealed that wt-ACAA2, unlike AASS, arrested LX2 cells at the G1/S transition (Fig. 4J), further underscoring the palmitoylation-dependent regulation of HSC proliferation.

Collectively, these data establish ACAA2 palmitoylation as a pivotal modulator of ferroptosis-driven HSC activation, offering a potential therapeutic target for MASLD-associated fibrosis.

### 3.7 ACAA2 deficiency attenuates liver fibrosis via GPX4-mediated ferroptosis

**Fig. 5:**
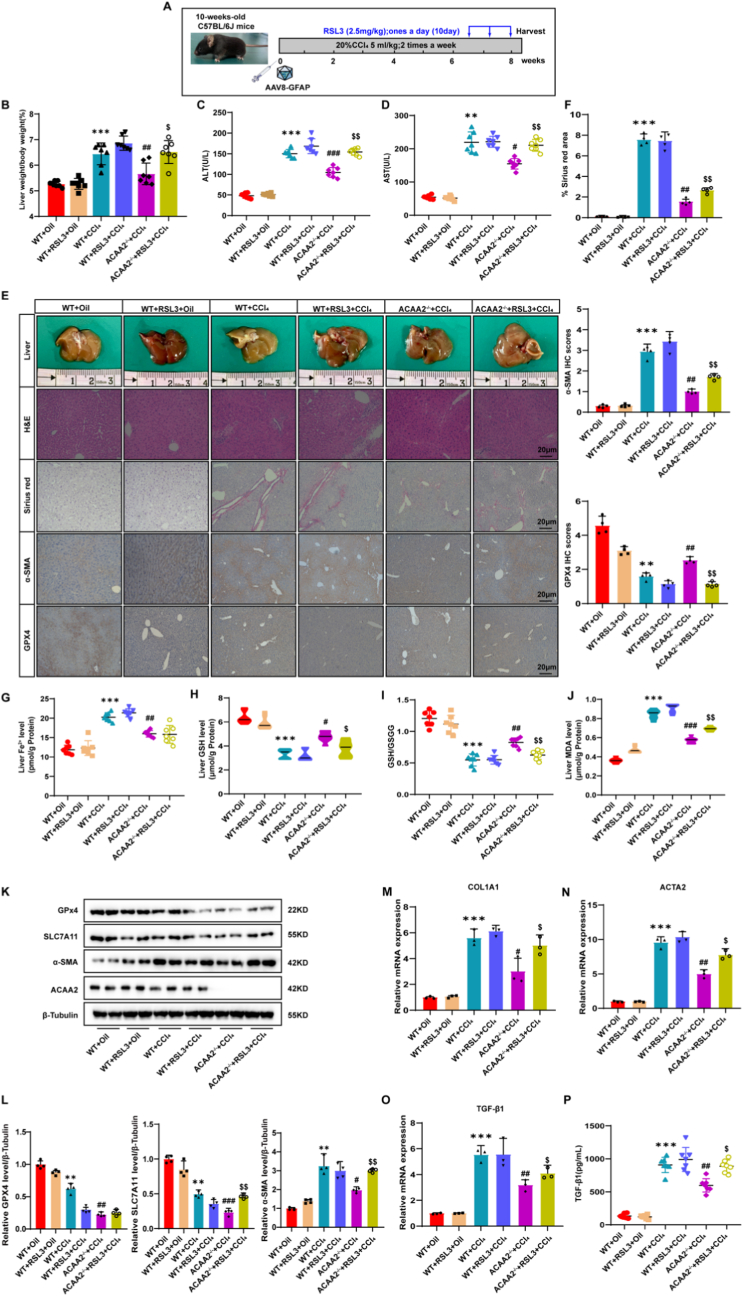
Deletion of ACAA2 reduces fibrosis in a CCl_4_ mouse model **(A)** Schematic overview of the experiments (n = 7/group). **(B)** Ratio of liver-to-body weight (n = 7/group). **(C-D)** Serum levels of ALT, AST (n = 7/group).**(E-F)** Representative images of gross liver morphology, H&E, Sirius red, immunohistochemical staining of α-SMA and GPX4, corresponding quantification of the positive IHC stained area (n = 4/group).**(G)** Level of liver Fe^2+^ tested using an iron assay kit (n = 7/group). **(H)** Level of GSH (n = 7/group). **(I)** Ratio of GSH to GSSG (n = 7/group). **(J)** Level of MDA (n = 7/group). **(K-L)** Representative immunoblots and quantitative histogram of GPX4, SLC7A11 and α-SMA in liver tissues of 6 groups of mice (n = 4/group). **(M-O)** The mRNA level of COL1A1,ACTA2 and TGF-β1 in liver tissues of 6 groups of mice(n = 3/group). **(P)** Serum levels of TGF-β1 were validated by ELISA (n = 7/group). Data presented are means ± SD. The differences among multiple groups were statistically analyzed by one-way ANOVA with Tukey's multiple comparisons test.∗*p* < 0.05, ∗∗*p < *0.01, ∗∗∗*p* < 0.001 vs WT + Oil; ^#^*p* < 0.05, ^##^*p* < 0.01,^###^*p* < 0.001 vs WT + CCl_4_; ^$^*p* < 0.05, ^$$^*p < *0.01, ^$$$^*p* < 0.001 vs ACAA2^−/−^+ CCl_4_. NS = no significance.

Having established that ACAA2 modulates hepatic stellate cell (HSC) activation through ferroptosis, we next investigated whether pharmacological induction of ferroptosis could reverse the protective effects of ACAA2 ablation. To this end, ***ACAA2***^−/−^mice were administered the ferroptosis inducer RSL3 (2.5 mg/kg, i.p., daily for 10 days) in both methionine-choline-deficient (MCD) diet-induced metabolic dysfunction-associated steatohepatitis (MASH) and carbon tetrachloride (CCl_4_)-induced liver fibrosis models (Fig. 5A and 7A).

Consistent with ferroptotic pathway activation, RSL3 treatment significantly suppressed GPX4 and SLC7A11 expression in both models (Fig. 5E–K,L, 7F). Strikingly, RSL3 administration abrogated the protective effects of ***ACAA2*** knockout, as evidenced by: Liver fibrosis progression: Increased liver weight, liver-to-body weight ratio, and histopathological collagen deposition (Sirius red staining and immunohistochemistry) (Fig. 5B–E,F, 7B,C,F,G); Hepatocellular injury: Elevated serum ALT and AST levels (Fig. 5C,D, 7D,E); Ferroptotic lipid peroxidation: Depletion of glutathione (GSH), reduced GSH/GSSG ratio, accumulation of malondialdehyde (MDA), and elevated redox-active iron (Fig. 5G–J, 7H–K). Furthermore, RSL3 restored the expression of HSC activation markers (mRNA) and TGF-β1 (protein), confirming that ferroptosis reinstates profibrotic signaling in *ACAA2*-deficient mice (Fig. 5 M − P, 7L–O).

Collectively, these findings demonstrate that ACAA2 deficiency exerts its antifibrotic effects through GPX4-dependent ferroptosis suppression, and its genetic ablation confers resistance to liver fibrosis—a protective mechanism that is negated upon ferroptosis induction.

### 3.8 ACAA2 overexpression in hepatocytes exacerbates steatosis, injury, and fibrogenesis

To investigate ACAA2's role in liver fibrosis, we overexpressed ACAA2 in hepatocytes (AAV8-TBG-*ACAA2*) or hepatic stellate cells (HSCs; AAV8-GFAP-*ACAA2*), with AASS overexpression as a control (AAV8-GFAP-*AASS*) (Figs. S12A, 6A, 8A).

Hepatocyte-specific ACAA2 overexpression induced: Macrosteatosis: Increased hepatic lipid vacuolization vs. controls (Fig. S12B and C).Fibrosis progression: Elevated Sirius red and α-SMA deposition (Fig. S12B and C).Hepatocyte injury: Increased serum ALT/AST (Fig. S12D) despite unchanged liver/body weight ratios in HFD-fed mice (Fig. S12E). Profibrogenic signaling: Upregulated TGF-β1 secretion, α-SMA protein, and transcription of *ACTA2*, *COL1A1*, and *TGF-β1* (Fig. S12F–H).

### 3.9 ACAA2 in HSCs promotes ferroptosis-dependent fibrosis, rescued by Lip-1

**Fig. 6:**
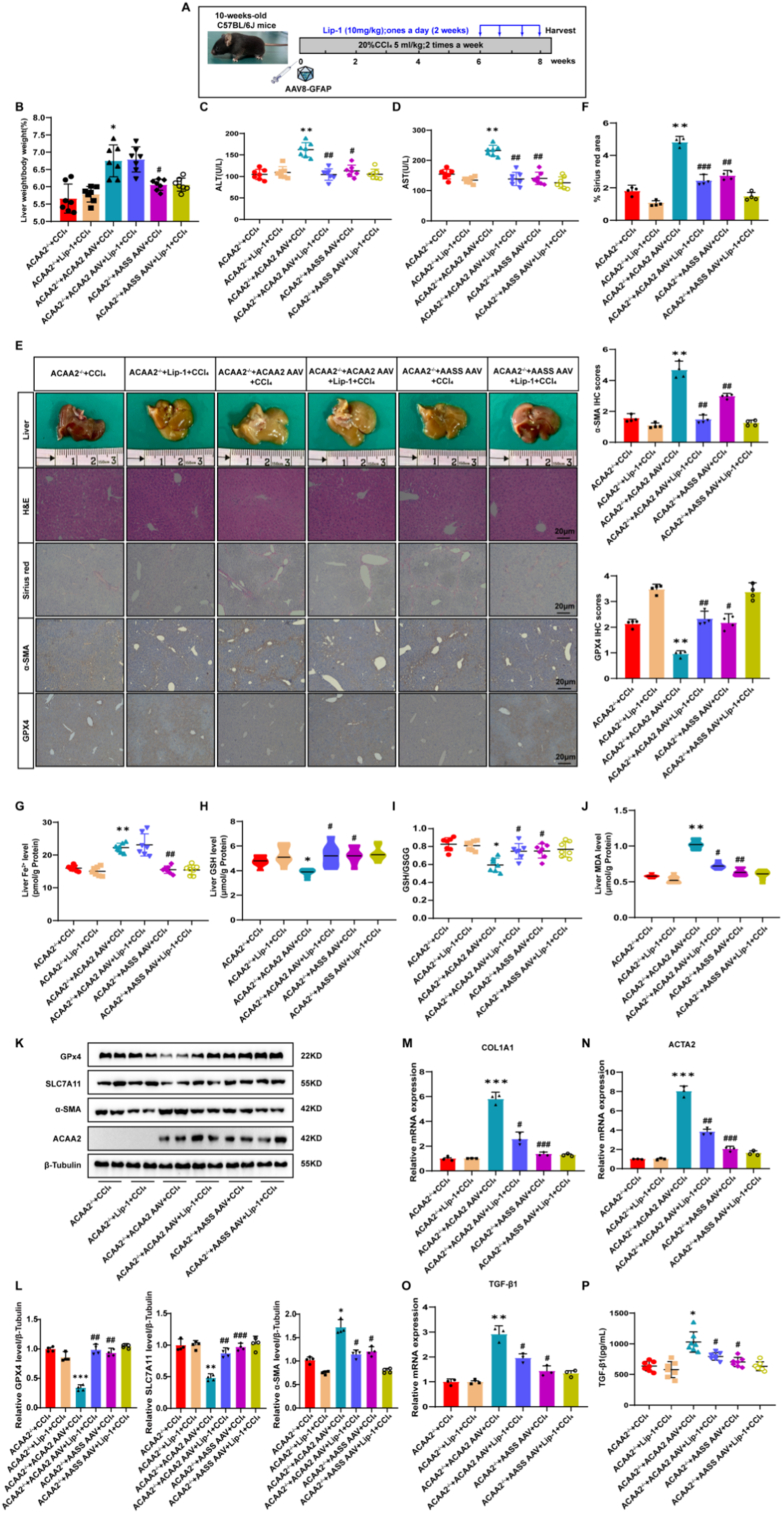
ACAA2 promotes fibrosis in a CCl_4_ mouse model **(A)** Schematic overview of the experiments (n = 7/group). **(B)** Ratio of liver-to-body weight (n = 7/group). **(C-D)** Serum levels of ALT, AST (n = 7/group).**(E-F)** Representative images of gross liver morphology, H&E, Sirius red, immunohistochemical staining of α-SMA and GPX4, corresponding quantification of the positive IHC stained area (n = 3/group). **(G)** Level of liver Fe^2+^ tested using an iron assay kit (n = 7/group). **(H)** Level of GSH (n = 7/group). **(I)** Ratio of GSH to GSSG (n = 7/group). **(J)** Level of MDA (n = 7/group). **(K-L)** Representative immunoblots and quantitative histogram of GPX4, SLC7A11 and α-SMA in liver tissues of 6 groups of mice (n = 4/group). **(M-O)** The mRNA level of COL1A1,ACTA2 and TGF-β1 in liver tissues of 6 groups of mice(n = 3/group). **(P)** Serum levels of TGF-β1 were validated by ELISA (n = 7/group). Data presented are means ± SD. The differences among multiple groups were statistically analyzed by one-way ANOVA with Tukey's multiple comparisons test.∗*p* < 0.05, ∗∗*p < *0.01, ∗∗∗*p* < 0.001 vs ACAA2^−/−^+ CCl_4_; ^#^*p* < 0.05, ^##^*p* < 0.01,^###^*p* < 0.001 vs ACAA2^−/−^+ ACAA2 AAV + CCl_4_. NS = no significance.

In both the CCl_4_-and MCD-induced fibrosis models, HSC-specific overexpression of ACAA2 triggered ferroptosis, evidenced by the depletion of GPX4 and SLC7A11 (Fig. 6E–K, L; 8F). Concurrently, ACAA2 overexpression accelerated fibrogenesis, as reflected by increased liver-to-body weight ratios, expanded Sirius Red–positive fibrotic areas, and elevated immunohistochemical staining for fibrotic markers (Fig. 6B–E, F; Fig. 8B, C, F, G).

**Fig. 7:**
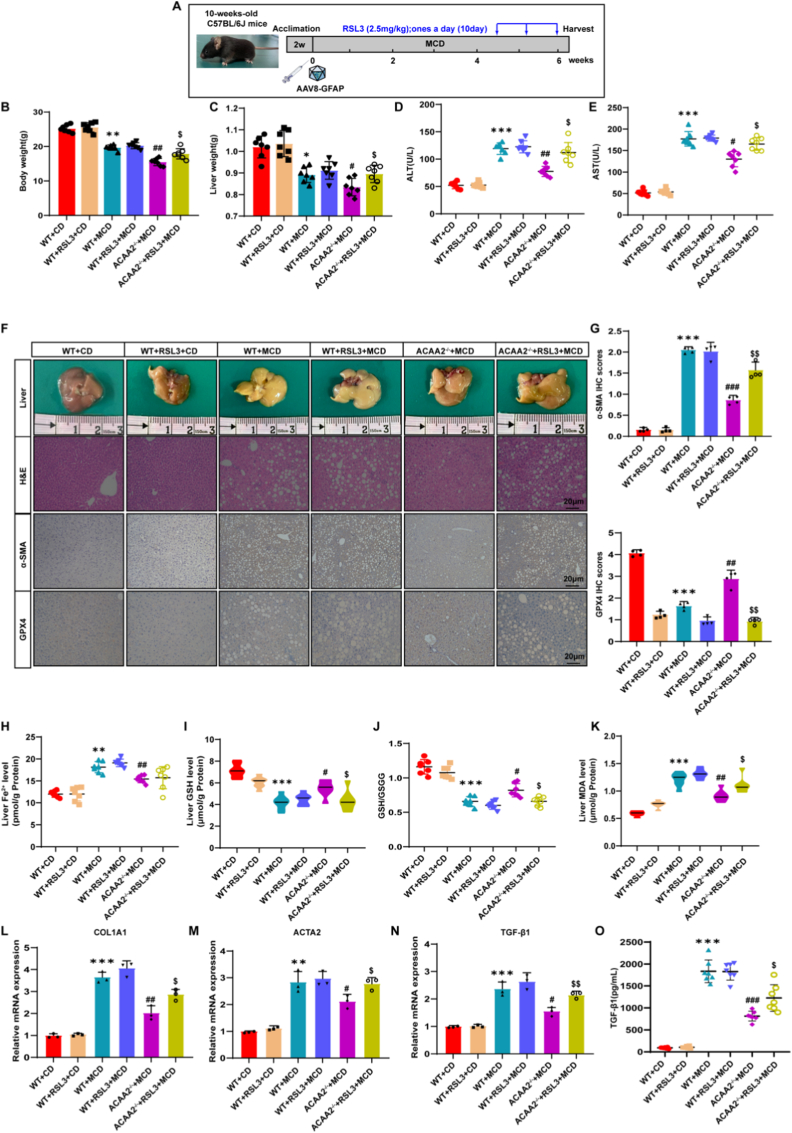
Deletion of ACAA2 reduces fibrosis in MASH mouse model **(A)** Schematic overview of the experiments (n = 7/group). **(B)** Body weight (n = 7/group). **(C)** Liver weight (n = 7/group). **(D-E)** Serum levels of ALT, AST (n = 7/group). **(F-G)** Representative images of gross liver morphology, H&E, Sirius red, immunohistochemical staining of α-SMA, corresponding quantification of the positive IHC stained area (n = 3/group). **(H)** Level of liver Fe^2+^ tested using an iron assay kit (n = 7/group). **(I)** Level of GSH (n = 7/group). **(J)** Ratio of GSH to GSSG (n = 7/group). **(K)** Level of MDA (n = 7/group). **(L-N)** The mRNA level of COL1A1,ACTA2 and TGF-β1 in liver tissues of 6 groups of mice(n = 3/group). **(O)** Serum levels of TGF-β1 were validated by ELISA (n = 7/group). Data presented are means ± SD. The differences among multiple groups were statistically analyzed by one-way ANOVA with Tukey's multiple comparisons test.∗*p* < 0.05, ∗∗*p < *0.01, ∗∗∗*p* < 0.001 vs WT + CD; ^#^*p* < 0.05, ^##^*p* < 0.01,^###^*p* < 0.001 vs WT + MCD; ^$^*p* < 0.05, ^$$^*p < *0.01, ^$$$^*p* < 0.001 vs ACAA2^−/−^+ MCD. NS = no significance.

**Fig. 8:**
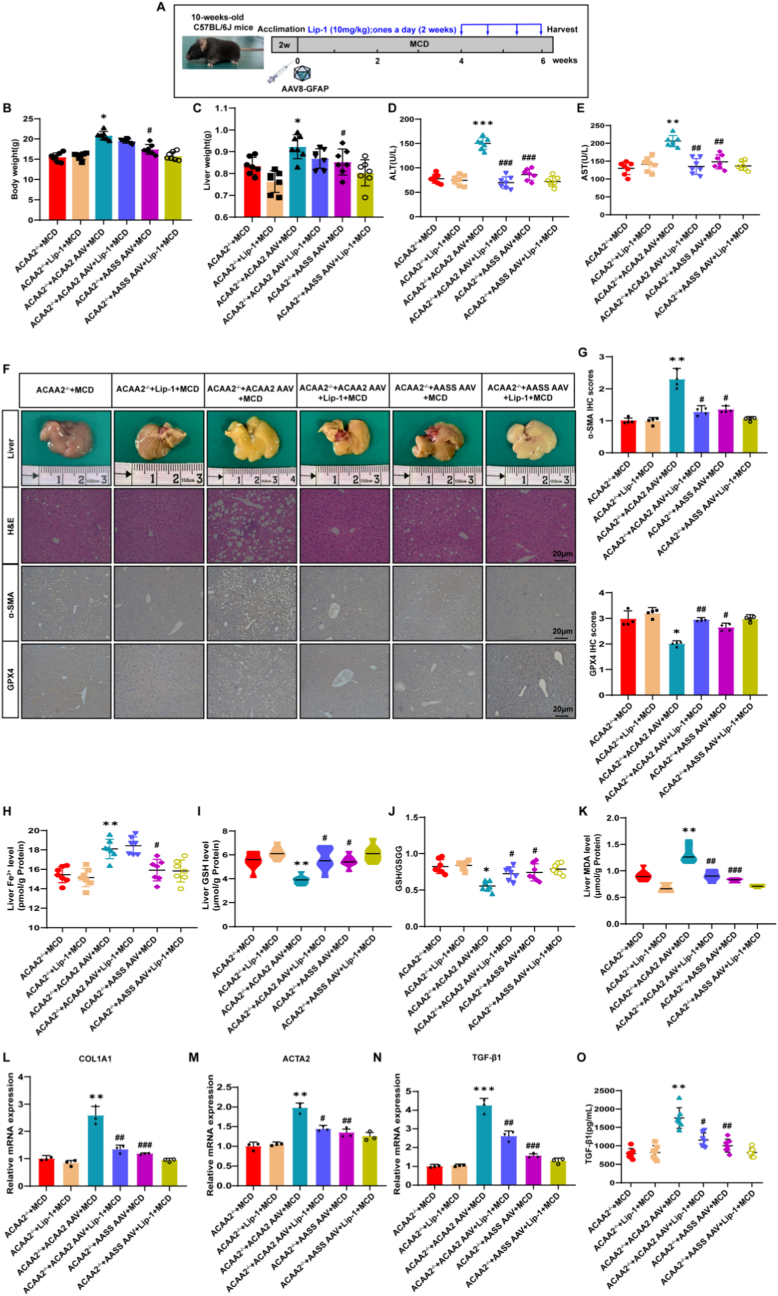
ACAA2 promotes fibrosis in MASH mouse model **(A)** Schematic overview of the experiments (n = 7/group). **(B)** Body weight (n = 7/group). **(C)** Liver weight (n = 7/group). **(D-E)** Serum levels of ALT, AST (n = 7/group). **(F-G)** Representative images of gross liver morphology, H&E, Sirius red, immunohistochemical staining of α-SMA, corresponding quantification of the positive IHC stained area (n = 3/group). **(H)** Level of liver Fe^2+^ tested using an iron assay kit (n = 7/group). **(I)** Level of GSH (n = 7/group). **(J)** Ratio of GSH to GSSG (n = 7/group). **(K)** Level of MDA (n = 7/group). **(L-N)** The mRNA level of COL1A1,ACTA2 and TGF-β1 in liver tissues of 6 groups of mice(n = 3/group). **(O)** Serum levels of TGF-β1 were validated by ELISA (n = 7/group). Data presented are means ± SD. The differences among multiple groups were statistically analyzed by one-way ANOVA with Tukey's multiple comparisons test.∗*p* < 0.05, ∗∗*p < *0.01, ∗∗∗*p* < 0.001 vs ACAA2^−/−^+ MCD; ^#^*p* < 0.05, ^##^*p* < 0.01,^###^*p* < 0.001 vs ACAA2^−/−^+ ACAA2 AAV + MCD. NS = no significance.

Lip-1 (ferroptosis inhibitor) treatment reversed these effects. Restored redox balance: Reduced iron overload, MDA, and GSH depletion while normalizing GSH/GSSG ratios (Fig. 6G–J; 8H–K); Attenuated injury/fibrosis: Lowered ALT/AST (Fig. 5C and D; 7D, E) and suppressed TGF-β1 and HSC activation markers (Fig. 6M–P; 8L–O). Specificity of ACAA2: AAV8-GFAP-*AASS* had negligible effects, confirming the palmitoylation of ACAA2's distinct profibrotic role.

ACAA2 promotes liver fibrosis by inducing ferroptosis in HSCs and cytokine release in hepatocytes, driving HSC activation and TGF-β1-mediated fibrogenesis. Its palmitoylation further underscores its mechanistic role in MASLD progression.

## 4 Discussion

Liver fibrosis represents a pivotal pathological stage in the progression of chronic liver diseases, including cirrhosis and hepatocellular carcinoma. Despite the well-established role of activated hepatic stellate cells (HSCs) in fibrogenesis, the molecular mechanisms driving their activation remain incompletely elucidated [27,28]. Our study identifies ACAA2—a key enzyme in fatty acid β-oxidation—as a critical regulator of HSC activation and ferroptosis, with direct implications for liver fibrosis. We demonstrate that ACAA2 expression is elevated in fibrotic liver tissue and that its inhibition attenuates HSC activation and ferroptosis in vitro. Furthermore, in vivo studies using CCl_4_-and MCD-induced liver injury models reveal that genetic suppression of ACAA2 exerts potent antifibrotic effects. Notably, we uncover ACAA2 palmitoylation as a novel post-translational modification that modulates HSC activation and ferroptosis, suggesting that targeted disruption of this modification may offer a promising therapeutic strategy for liver fibrosis, particularly in MASLD, for which no approved therapies currently exist.

Previous studies have implicated ACAA2 in diverse hepatic processes. Elevated ACAA2 expression has been reported in the fatty liver of obese patients, and its upregulation has been linked to protection against BNIP3-mediated apoptosis and acetaminophen-induced hepatotoxicity [29]. Conversely, CAND1 was shown to mitigate MASLD by suppressing Cullin1/FBXO42-mediated ACAA2 degradation [30]. However, the role of ACAA2 palmitoylation in liver fibrosis and ferroptosis remained unexplored. Our findings reveal that ACAA2 overexpression exacerbates HSC activation and ferroptosis, whereas ACAA2 knockout attenuates fibrosis and improves liver function in injury models. Intriguingly, ACAA2 participates in fatty acid oxidation—a process previously associated with HSC quiescence. While inhibition of ATP-citrate lyase (ACLY) has been shown to reduce hepatic lipid accumulation and fibrosis via enhanced fatty acid oxidation [31], its role in ferroptosis regulation is highly context-dependent, particularly in HSCs. In fibrotic livers, excessive iron accumulation markedly enhances lipid peroxidation and ferroptotic sensitivity, even when FAO is elevated. Enhanced FAO also increases mitochondrial oxidative phosphorylation and ROS production, which can serve as upstream signals to amplify ferroptotic lipid peroxidation under metabolic stress. Moreover, accelerated lipid droplet mobilization during HSC activation may replenish PUFA-containing phospholipids, expanding the substrate pool for ferroptosis despite increased fatty acid oxidation. Our study underscores the need for further investigation into how metabolic reprogramming, particularly via ACAA2, influences fibrotic progression.

Mechanistically, we demonstrate that ACAA2 inhibition suppresses primary HSC activation, as evidenced by reduced α-SMA expression and downregulation of fibrogenic and ferroptosis-related transcripts. These findings suggest that ACAA2 blockade may reverse HSC activation and mitigate ferroptosis—a process whose role in fibrosis remains contentious. While iron overload has long been implicated in fibrogenesis, conflicting reports exist regarding ferroptosis modulation. For instance, erastin was found to exacerbate diet-induced MASH, whereas other studies reported that erastin and sorafenib ameliorated fibrosis via autophagy regulation [32,33]. Our data align with Tong and Bi's work [34,35], showing that Lip-1 (a ferroptosis inhibitor) attenuates fibrosis, whereas RSL3 (a ferroptosis inducer) exacerbates it in CCl_4_-and MCD-treated models.

At the molecular level, we establish that ACAA2 regulates ferroptosis via the AMPK/SLC7A11/GPX4 axis. Overexpression of ACAA2 in HSCs suppresses phosphorylated AMPK, SLC7A11, and GPX4 levels, whereas ACAA2 knockdown has the opposite effect. These observations suggest that ACAA2 promotes ferroptosis and HSC activation, at least in part, by inhibiting AMPK signaling.

Crucially, we identify ACAA2 palmitoylation as a druggable target. Pharmacological inhibition of palmitoylation (using 2-BP) or mutation of ACAA2 palmitoylation sites (AA-SS) attenuates ferroptosis, inflammation, and fibrogenesis. Although 2-BP is a broad-spectrum palmitoylation inhibitor, our findings highlight the need for selective ACAA2 palmitoylation inhibitors to optimize therapeutic efficacy while minimizing off-target effects.

Our study elucidates a previously unrecognized role for ACAA2 in liver fibrosis, mediated through its regulation of HSC activation, ferroptosis, and metabolic reprogramming. We propose a model wherein liver injury upregulates ACAA2 expression and palmitoylation in hepatocytes, which in turn enhances ACAA2 levels in HSCs, promoting their activation via transcriptional and ferroptosis-related pathways. Additionally, ACAA2 amplifies cytokine crosstalk between hepatocytes and HSCs, further driving fibrogenesis. These findings position ACAA2 inhibition—particularly via disruption of palmitoylation—as a compelling therapeutic strategy for MASLD and liver fibrosis/cirrhosis. Future studies should focus on developing isoform-specific ACAA2 modulators and delineating the broader metabolic consequences of ACAA2 targeting in preclinical models.

## 5 Limitations of study

However, our studies also have some limitations. First, CCl_4_ and MCD diet cannot fully induce chronic liver fibrosis. Although it has been reported that CCl_4_ and MCD diet can induce the mouse model of liver fibrosis, the etiology of liver fibrosis is mostly caused by hepatitis B virus infection, alcohol and/or drug use, or primary or secondary forms of cholestasis. Thus, the MCD diet and CCl_4_-induced mouse model of chronic liver injury only exhibits part of the clinical pathological phenotypes. Thus, evaluation of ACAA2 expression should be evaluated in other mouse models of chronic liver injury, as well as in more clinical samples with a better-defined etiology of liver fibrosis.Second,Our study reveals that ACAA2 regulation of HSC activation, ferroptosis, and metabolic reprogramming during MASLD progression. However, what remains unknown is whether macrophage ACAA2 in MASLD livers participate in macrophage functions traditionally ascribed to this receptor, namely, efferocytosis, dampening of inflammation, and resolution of inflammation. Finally, our study shows that ferroptosis and palmitoylation of ACAA2 is increased in fibrotic livers, but the mechanisms of these changes needs to be determined.

## Financial support

This work was supported by the 10.13039/501100001809National Natural Science Foundation of China(No. 31771310 to X.Y., No.41871210 to L.W.and No.: 32400595 to F.W.).

## CRediT authorship contribution statement

**Jianxiong Han:** Conceptualization, Data curation, Investigation, Writing – original draft. **Zhongkang Yan:** Data curation, Formal analysis. **Zhiran Sun:** Data curation, Formal analysis. **Wenyuan Dang:** Data curation, Formal analysis. **Bao Li:** Formal analysis. **Shuangshuang Li:** Data curation, Formal analysis. **Xinru Lv:** Data curation, Formal analysis. **Lin Ni:** Data curation, Formal analysis. **Anyuan He:** Data curation, Formal analysis. **Pengying Gu:** Resources. **Feifei Wang:** Conceptualization, Funding acquisition, Writing – original draft. **Lili Wang:** Funding acquisition, Writing – original draft. **Xingyuan Yang:** Conceptualization, Funding acquisition, Project administration, Writing – review & editing.

## Declaration of competing interest

The authors who have taken part in this study declare that they do not have anything to disclose regarding funding or conflict of interest with respect to this manuscript. Please refer to the accompanying coi-disclosure forms for further detail.

## Data Availability

No data was used for the research described in the article.

## List of Abbreviations

AMPK, AMP-activated protein kinase; ACTA2/α-SMA, α-smooth muscle actin; HSC, hepatic stellate cell; ALT, alanine aminotransferase; AST, aspartate transaminase; CCl_4_, carbon tetrachloride; Col1A1, collagen type 1 alpha 1; DAPI,40,6-diamidino-2-phenylindole; HCC, hepatocellular carcinoma; LD, Lipid droplet; GPX4, Glutathione peroxidase 4; HFD, high-fat diet; MASLD, metabolic dysfunction-associated steatotic liver disease; MASH, metabolic dysfunction–associated steatohepatitis; siRNA, small interfering RNA; TGF-β1, transforming growth factor beta-1; MCD, Methionine-Choline Deficient; acetyl CoA acyltransferase 2; T2DM, type 2 diabetes mellitus; CoA, coenzyme A; AAV, adeno-associated virus; IHC, immunohistochemistry; PTMs, post-translational modifications.
