# Supplementary material for: Acetyl-CoA acyltransferase 2 palmitoylation drives liver fibrosis by inducing hepatic stellate cell ferroptosis
Source: Redox Biol. 2026 Jan 17;90:104035. doi: 10.1016/j.redox.2026.104035 (PMC12859212; doi:10.1016/j.redox.2026.104035)
Supplement: Multimedia component 1 [file mmc1.docx]

**Supplementary figures and table**

**
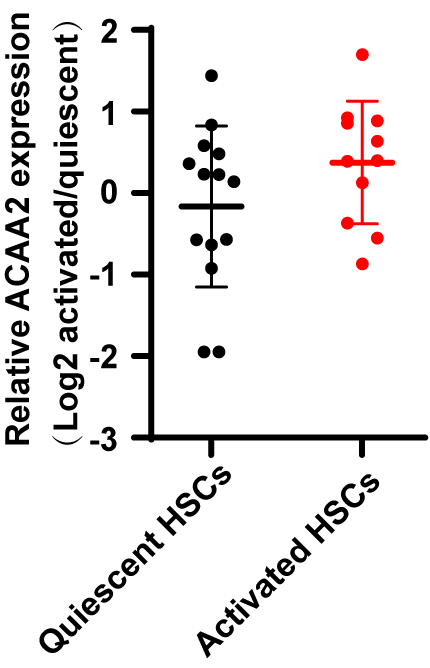
**

**Figure S1. Relative expression of ACAA2**

In human quiescent ( n=14 )and activated HSCs( n=11 ), the relative expression of ACAA2 was upregulated (GSE158723). Data were shown as log2-ratio of the individual value of activated samples to the mean value of quiescent samples.log₂-ratio = +0.54


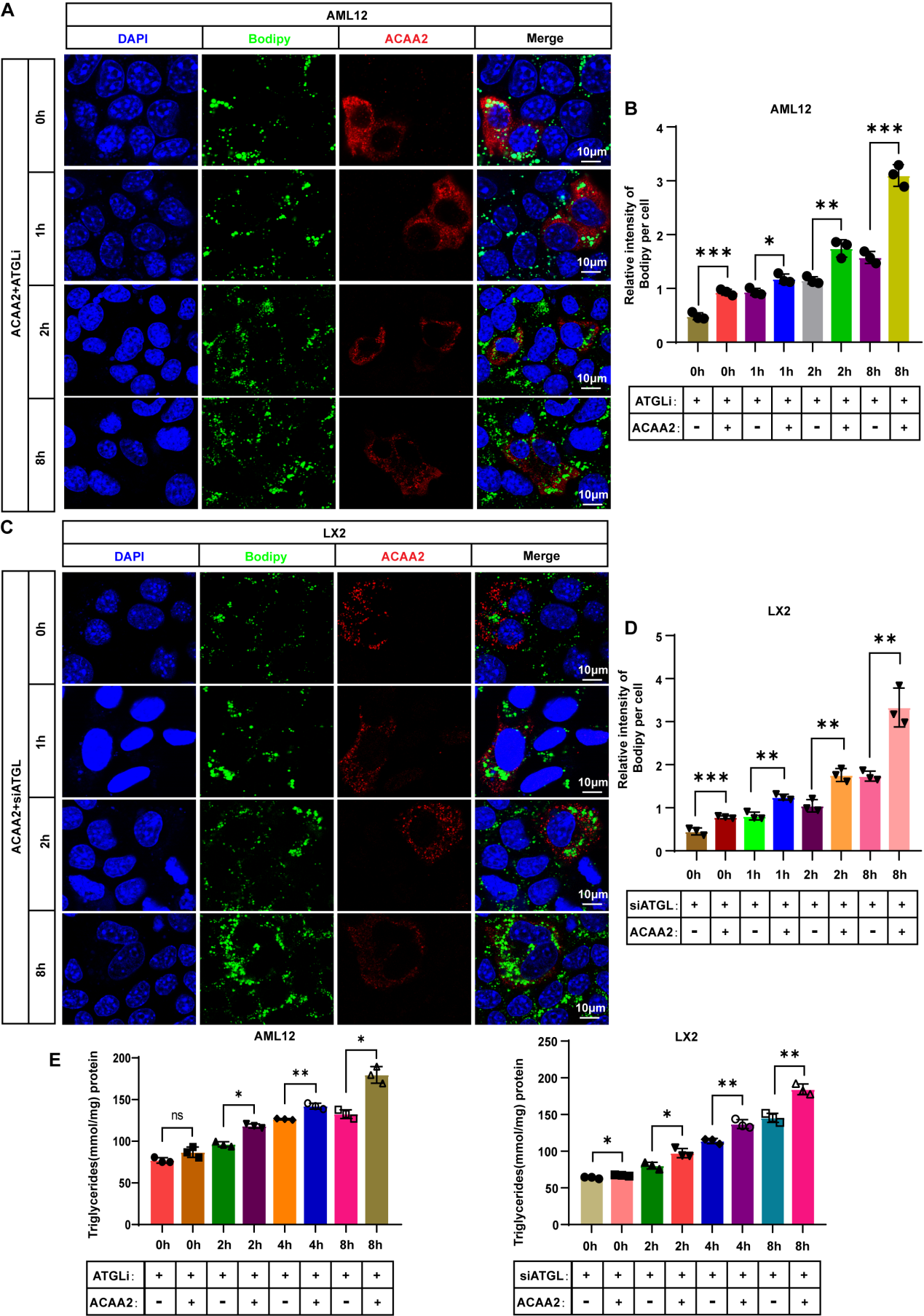


**Figure S2. ACAA2 Overexpression Modulates FFA uptake and Lipid Droplet Accumulation**

(A–C) ACAA2 overexpression significantly alters lipid droplet (LD) dynamics in OA/PA treated cells. (B, D) Quantification of LD accumulation under control and ACAA2-overexpressing conditions (n = 3 biological replicates per group). (E) Temporal analysis of triglyceride (TG) content in hepatocytes and hepatic stellate cells (HSCs) treated with OA/PA. Concurrent ACAA2 overexpression and ATGL inhibition synergistically enhanced TG accumulation over an 8-hour time course. Data presented are means ± SD. The differences among multiple groups were statistically analyzed by one-way ANOVA with Tukey's multiple comparisons test.**p* < 0.05, ***p*< 0.01, ****p* < 0.001 . NS = no significance.

**
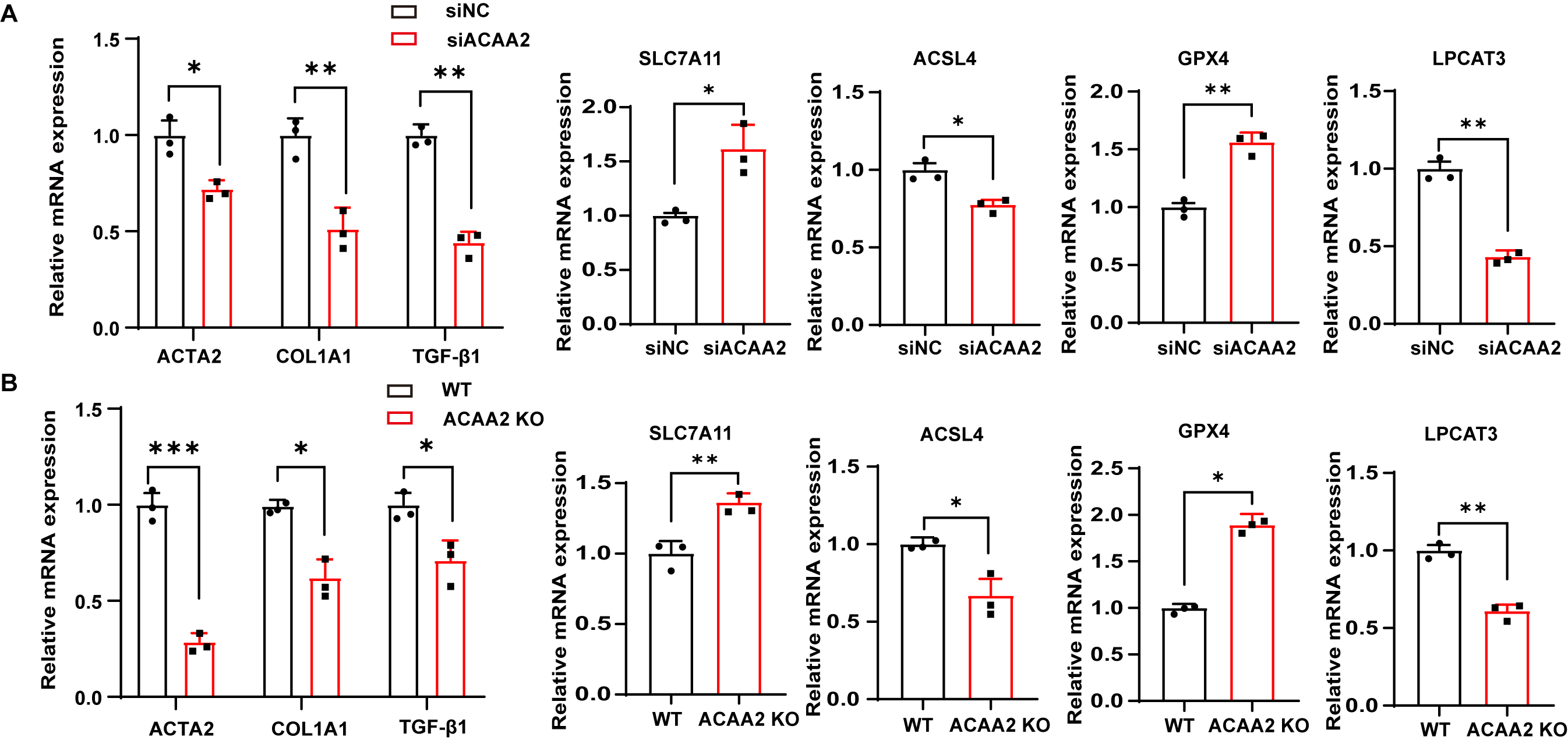
**

**Figure S3. Genetic Inhibition of ACAA2 Attenuates Ferroptosis and Hepatic Stellate Cell Activation**

(A) siRNA-mediated knockdown of ACAA2 significantly reduces mRNA levels of ferroptosis-associated genes (e.g., ACSL4, PTGS2) and markers indicative of HSC activation (e.g., ACTA2, COL1A1) and fibrogenesis in human LX-2 hepatic stellate cells.(B) Consistent with (A), CRISPR-generated ACAA2 knockout in primary HSCs demonstrates concordant suppression of ferroptosis-related transcripts and profibrotic activation markers.Data represent mean ± SEM (n = 3 biological replicates). Statistical significance was determined by two-tailed Student’s t-test (*P < 0.05, **P < 0.01, ***P < 0.001).


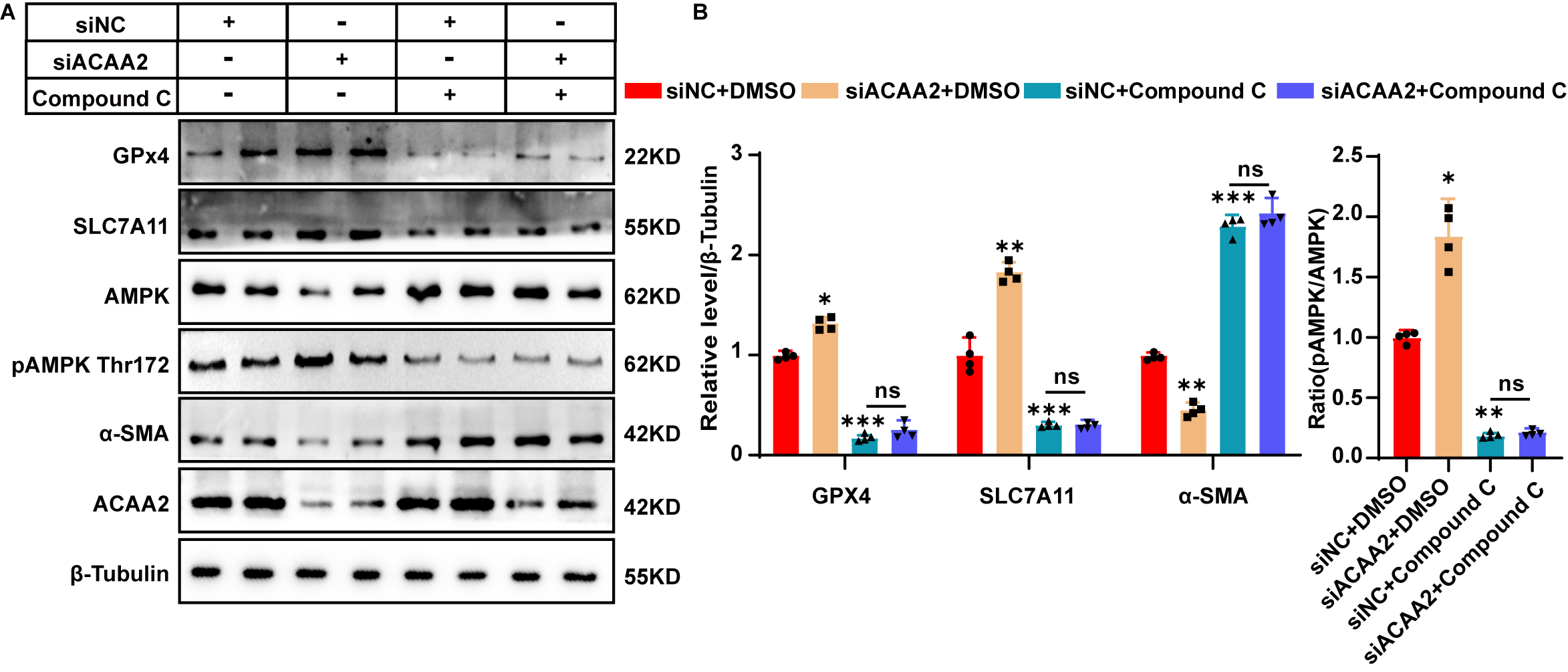


**Figure S4. Inhibition of ACAA2 is ineffective in the Presence of AMPK Signaling Blockade**

(A) Representative immunoblots of related protein following transfection with either non-targeting control siRNA (siNC) or ACAA2-targeting siRNA (siACAA2) under AMPK inhibition (10 μM Compound C).(B) Band intensity was quantified by densitometry. Data presented are means ± SD (n = 4 biological replicates). The differences among multiple groups were statistically analyzed by one-way ANOVA with Tukey's multiple comparisons test.**p* < 0.05, ***p*< 0.01, ****p* < 0.001 vs siNC+DMSO. NS = no significance.


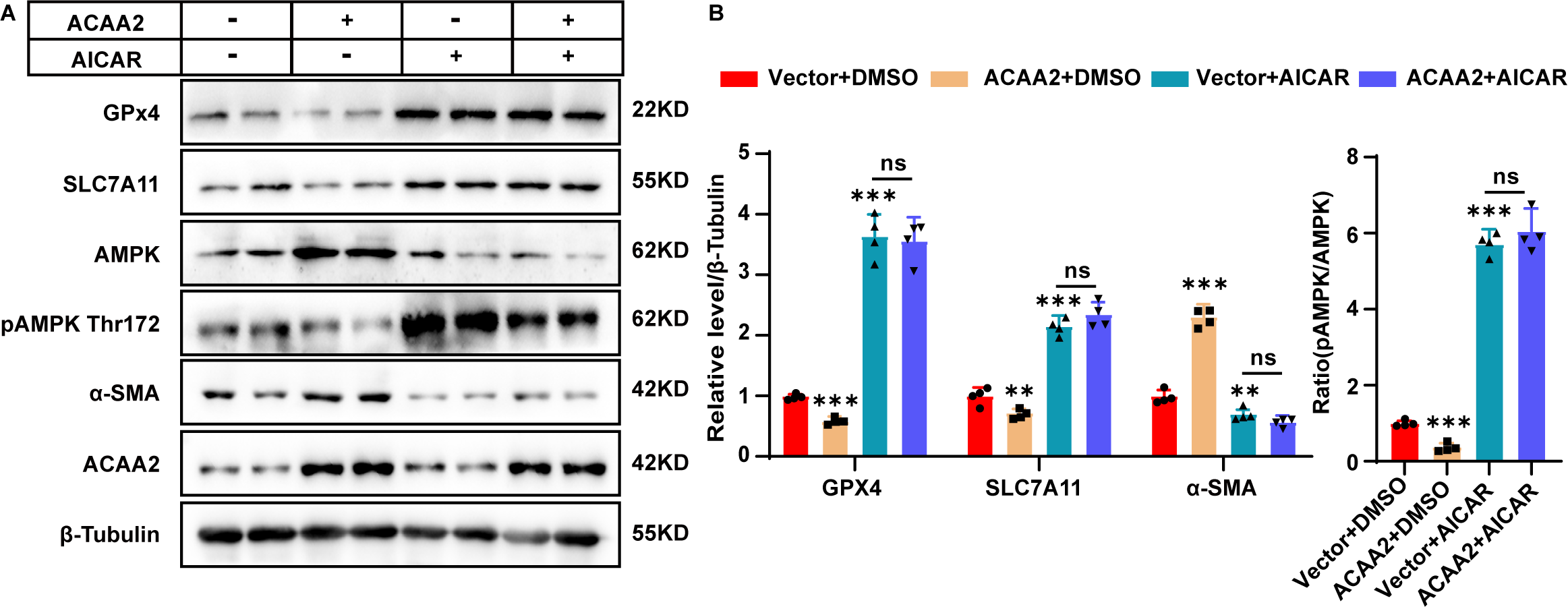


**Figure S5. The ineffectiveness of overexpression ACAA2 when the AMPK signal is activated**

(A) Representative immunoblots of related protein following transfection with either Vector or ACAA2 under AMPK activator (1 mM AICAR ).(B) Band intensity was quantified by densitometry. Data presented are means ± SD (n = 4 biological replicates). The differences among multiple groups were statistically analyzed by one-way ANOVA with Tukey's multiple comparisons test.**p* < 0.05, ***p*< 0.01, ****p* < 0.001 vs Vector+DMSO. NS = no significance.


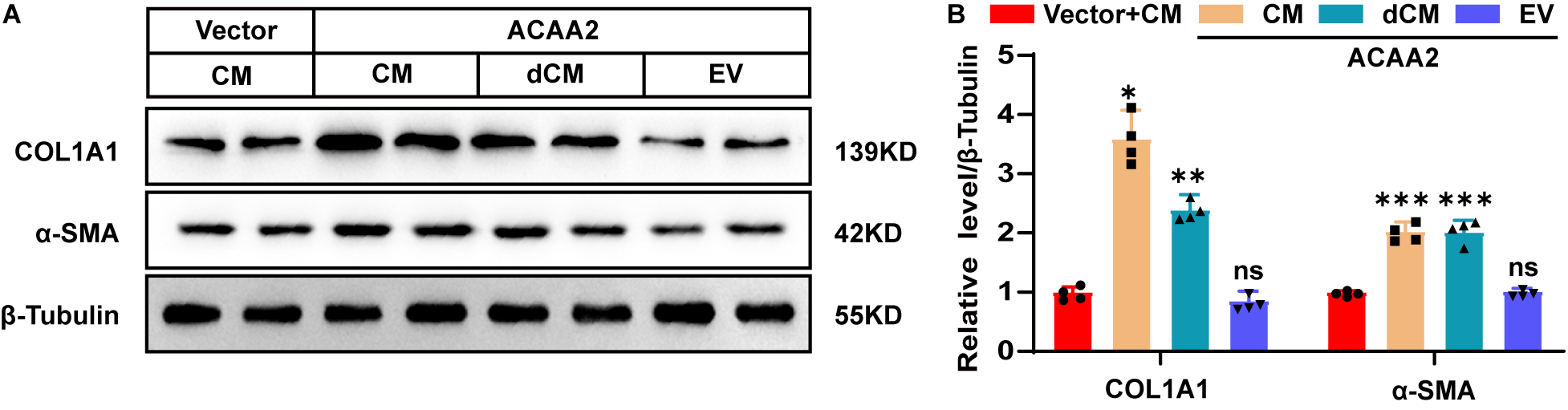


**Figure S6. The contributions of soluble factors and vesicular in conditioned medium to LX-2 cells**

(A)Treat LX-2 cells with CM (conditioned medium), dCM (EV-depleted conditioned medium), and EV (extracellular vesicles), and detect changes in α-SMA and COL1A1.(B) Band intensity was quantified by densitometry.The differences among multiple groups were statistically analyzed by one-way ANOVA with Tukey's multiple comparisons test (n = 4 biological replicates).**p* < 0.05, ***p*< 0.01, ****p* < 0.001 vs Vector+CM. NS = no significance.


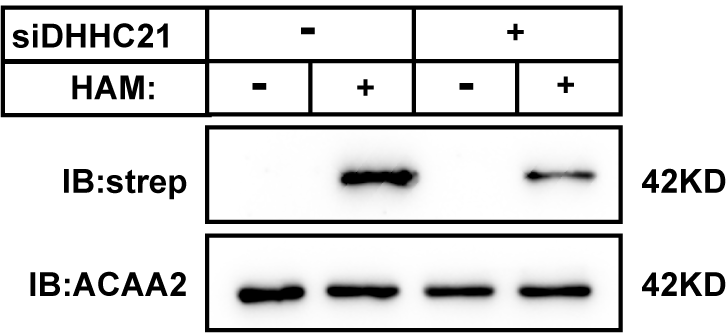


**Figure S7. Knockdown DHHC21 inhibits palmitoylation of ACAA2**

Control and DHHC21 knockdown LX-2 cells harvested for IP-ABE analysis.


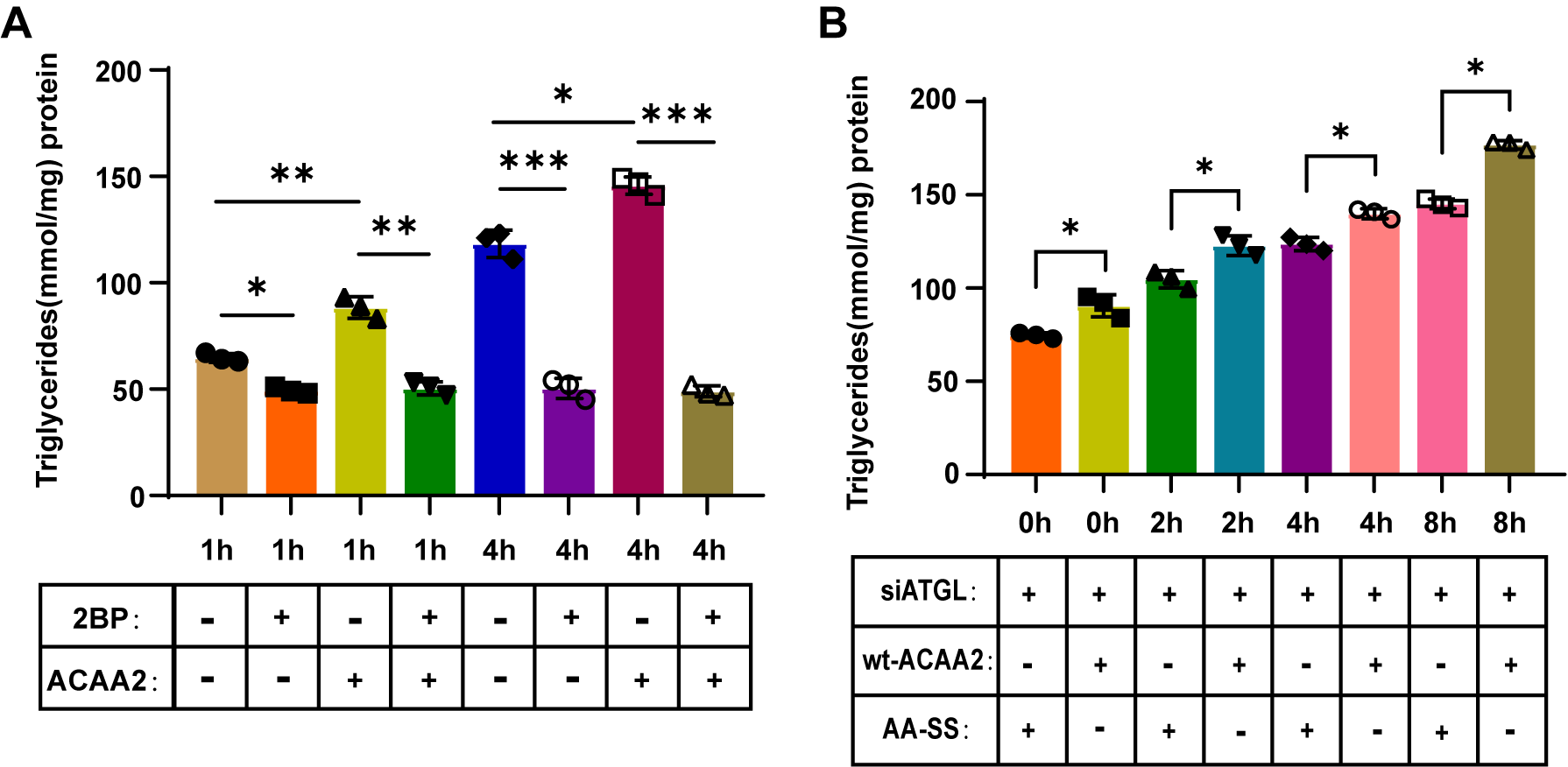


**Figure S8. Inhibition of ACAA2 Palmitoylation Attenuates Hepatic Lipid Accumulation**

(A) LX-2 cells overexpressing ACAA2 were treated with the palmitoylation inhibitor 2-bromopalmitate (2-BP, 100 μM) and OA/PA.(B) LX-2 cells transfected with either wild-type ACAA2 (wt-ACAA2) or a palmitoylation-deficient mutant (AA-SS) were exposed to OA/PA for 0-8 hr. Intracellular triglyceride (TG) levels were quantified via enzymatic assay.Data presented are means ± SD. The differences among multiple groups were statistically analyzed by one-way ANOVA with Tukey's multiple comparisons test.**p* < 0.05, ***p*< 0.01, ****p* < 0.001 . NS = no significance.


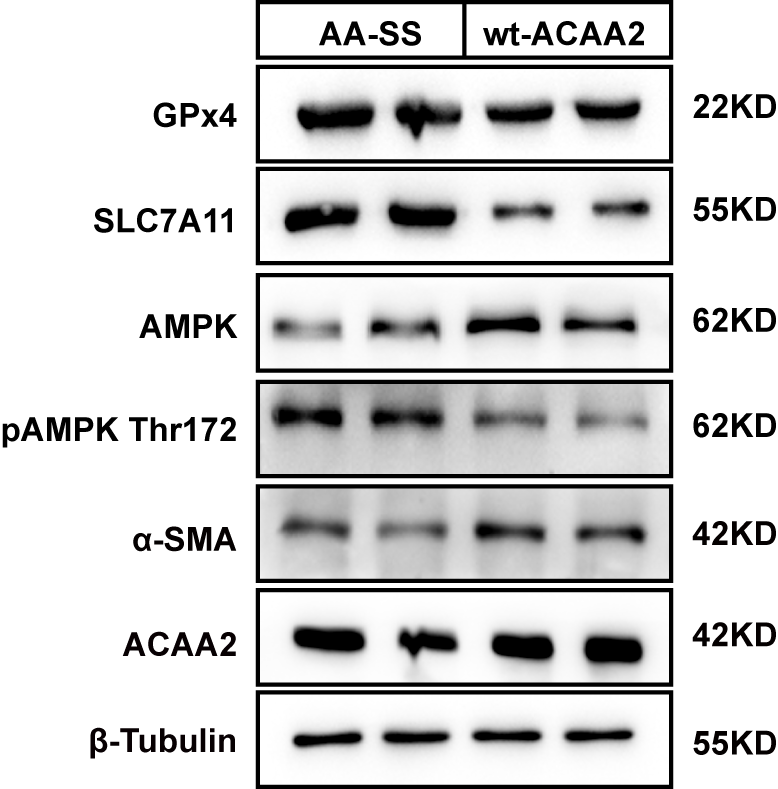


**Fig. S9. Protein expression profiles in AA-SS and wt-ACAA2-HSCs**

Representative immunoblots demonstrating relative expression levels of AMPK, phosphorylated AMPK (pAMPK), GPX4, SLC7A11, and α-SMA in LX2 cells. Quantification data are presented as mean ± SEM (n = 4 biological replicates). Statistical significance was determined by two-tailed Student’s t-test (*P < 0.05, **P < 0.01, ***P < 0.001).


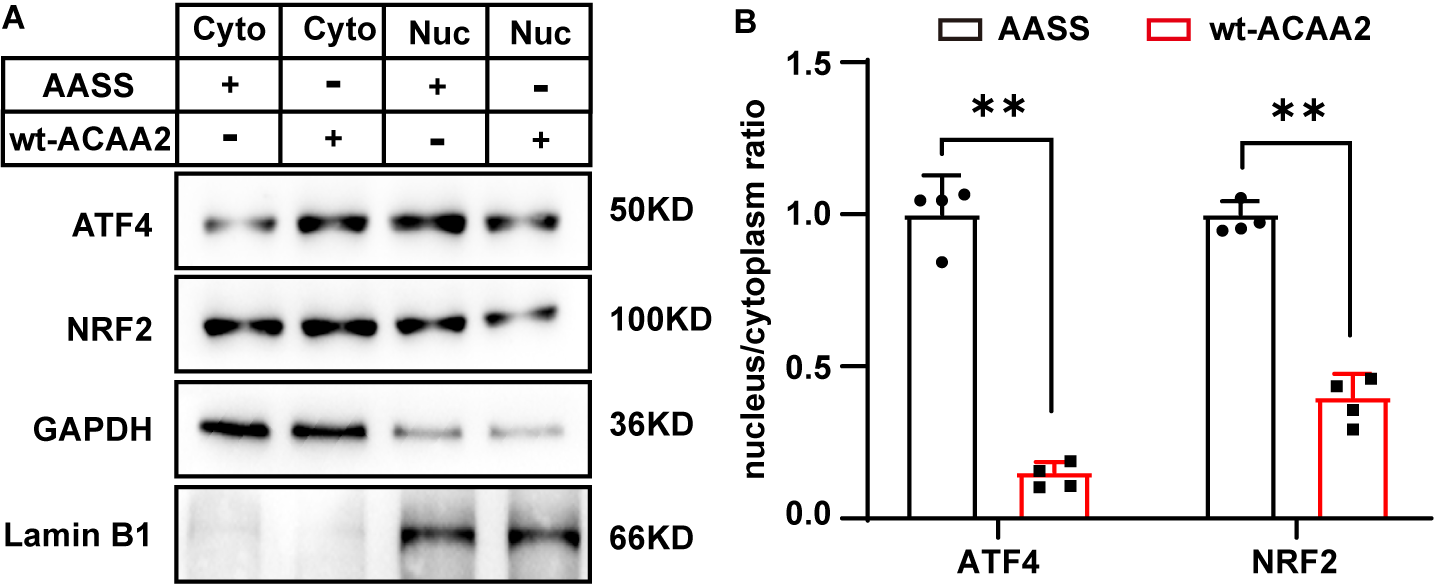


**Figure S10.The palmitoylation of ACAA2 affects the subcellular localization of ATF4 and NRF2**

1. Cytoplasmic and nuclear fractions were isolated from OA/PA-treated LX-2 cells overexpressing AASS and wt-ACAA2, and the expression levels of ATF4 and NRF2 in these fractions were examined by Western blotting. (B) Band intensity was quantified by densitometry. Data presented are means ± SD (n = 3 biological replicates). NS = not significant. **p* < 0.05; ***p* < 0.01.****p* < 0.001, *t*-test.

**
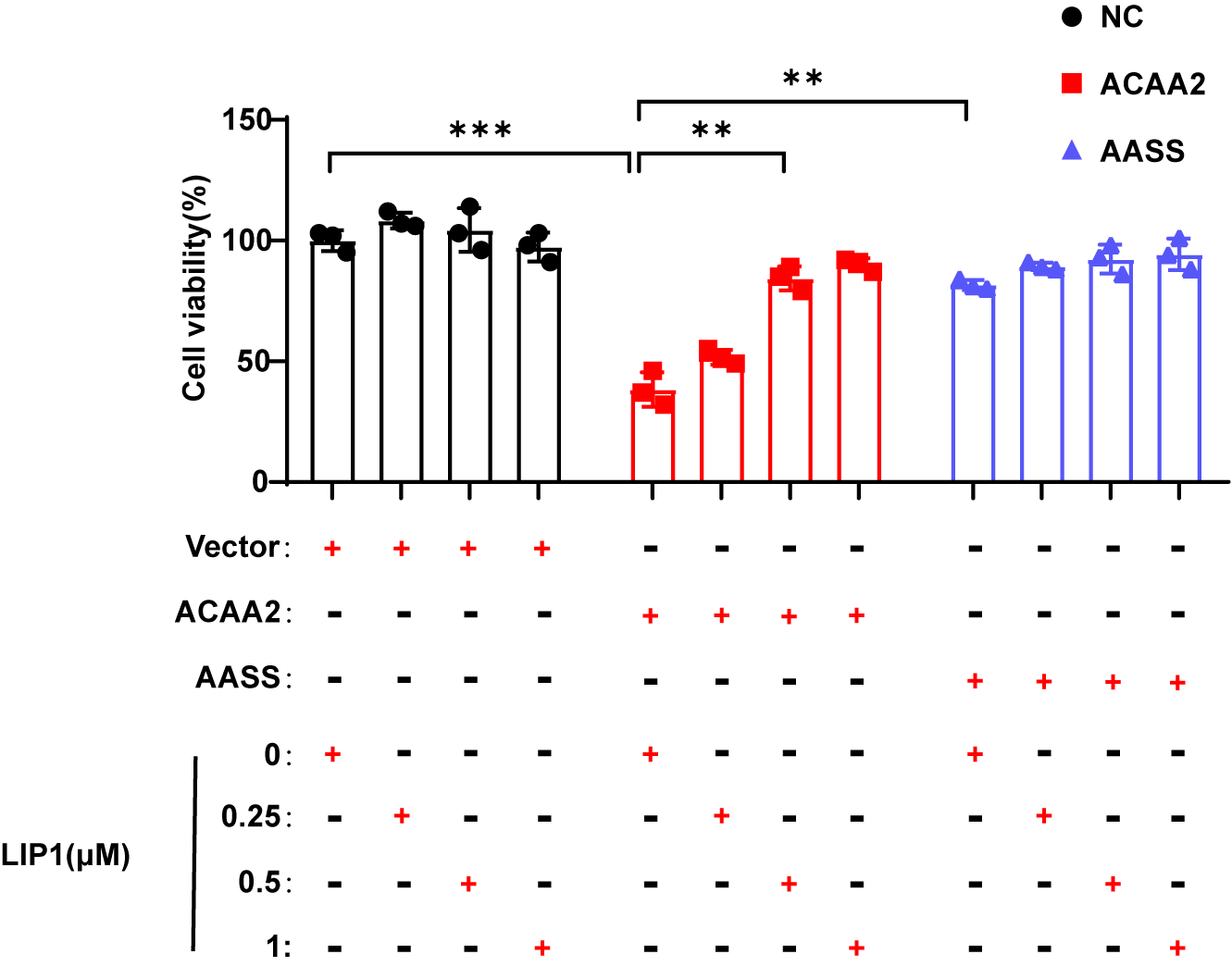
**

**Figure S11. Ferroptosis Inhibitor Liproxstatin-1 (LIP1) Attenuates Cytotoxicity in LX2 Cells**

Dose-response analysis of LX2 cell viability following treatment with Liproxstatin-1 (0–1 μM) for 24 hr. Data presented are means ± SD. The differences among multiple groups were statistically analyzed by one-way ANOVA with Tukey's multiple comparisons test.**p* < 0.05, ***p*< 0.01, ****p* < 0.001 . NS = no significance.


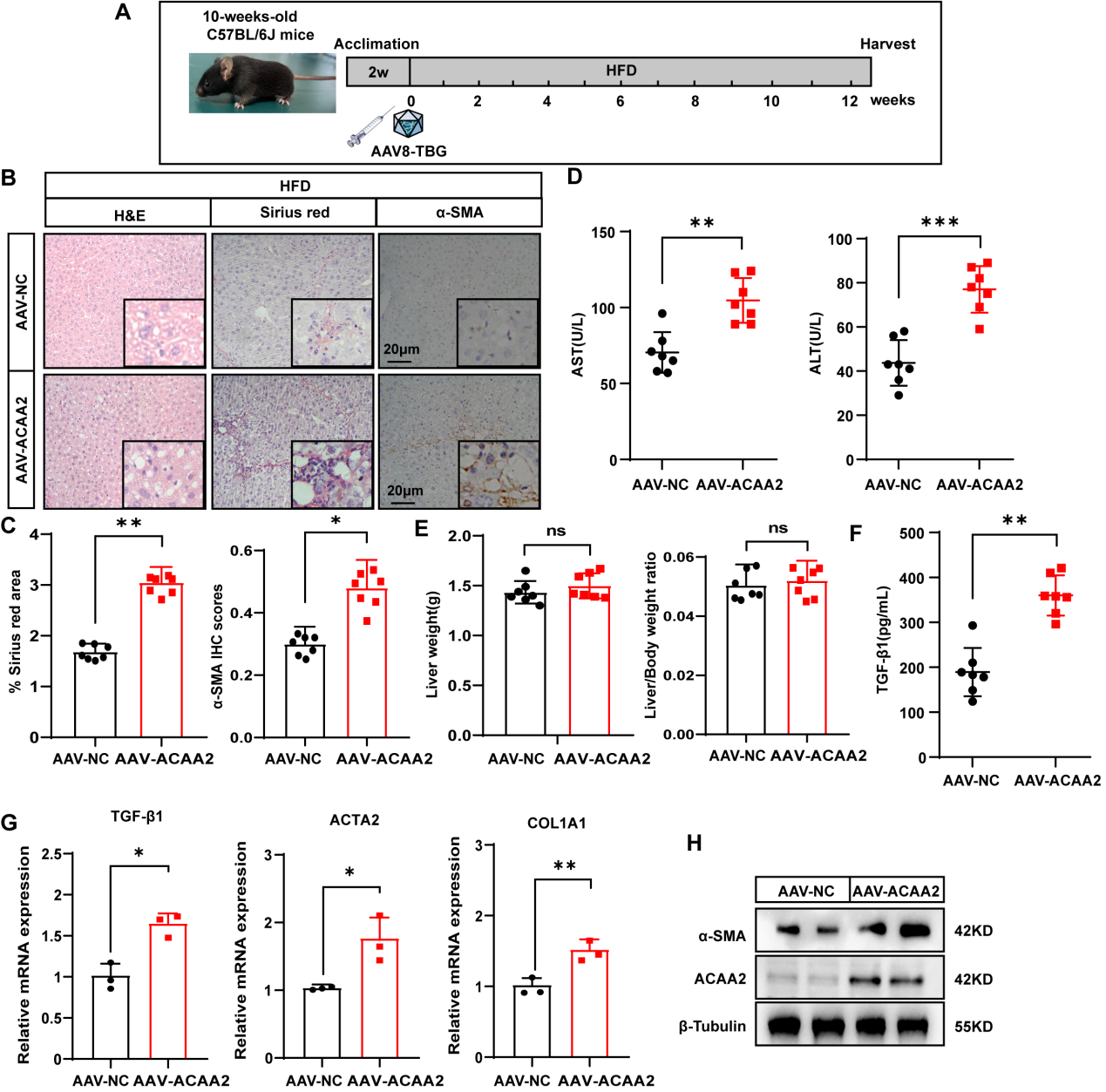


**Figure S12. Hepatocyte-Specific ACAA2 Exacerbates Steatosis and Fibrosis in HFD-Fed Mice**

(A) Schematic of experimental design: ACAA2 overexpression in hepatocytes was evaluated in a high-fat diet (HFD)-induced metabolic dysfunction-associated fatty liver disease (MAFLD) murine model.(B) Representative photomicrographs of H&E (steatosis), Sirius Red (collagen deposition), and α-SMA (hepatic stellate cell activation) staining in liver sections (n=3 biologically independent samples per group). Scale bars: 20 µm.(C) Quantification of fibrotic area (% total tissue) by Sirius Red staining and α-SMA+ immunoreactivity by immunohistochemistry (n=3).(D) Circulating ALT and AST levels (U/L), indicators of hepatocellular injury (n=7).(E) Absolute liver weight and liver-to-body weight ratio (%) in WT versus AAV-TBG-ACAA2 mice.(F) Serum TGF-β1 concentrations measured by ELISA (pg/mL).(G) qRT-PCR analysis of fibrogenic gene expression (Col1a1, Acta2, Tgfb1) in HFD-fed AAV-NC (n=3) versus AAV-ACAA2 (n=3) mice. Data normalized to Gapdh.(H) Western blot analysis of α-SMA protein expression in liver lysates (n=4).Data represent mean ± SD. Statistical significance determined by two-tailed Student’s t-test: *P < 0.05, **P < 0.01, ***P < 0.001; NS, not significant.

**Supplementary Table1.** Real-time polymerase chain reaction primers.

| Gene | Primer sequence (5′ to 3′) |
| --- | --- |
| Human *CXCL10-F* | CCAAGTGCTGCCGTCATTTTC |
| Human *CXCL10-R* | GGCTCGCAGGGATGATTTCAA |
| Human *CXCL1-F* | CTGGGATTCACCTCAAGAACATC |
| Human *CXCL1-R* | CAGGGTCAAGGCAAGCCTC |
| Human *CXCL2-F* | CCAACCACCAGGCTACAG |
| Human *CXCL2-R* | GCGTCACACTCAAGCTCTG |
| Human *IL-8-F* | GGCACAAACTTTCAGAGACAGCAG |
| Human *IL-8-R* | GTTTCTTCCTGGCTCTTGTCCTAG |
| Human *TGF-β1-F* | GGCCAGATCCTGTCCAAGC |
| Human *TGF-β1-R* | GTGGGTTTCCACCATTAGCAC |
| Human *ACTA2-F* | CGTGGCTATTCCTTCGTTAC |
| Human *ACTA2-R* | TGCCAGCAGACTCCATCC |
| Human *COL1A1-F* | CAGCCGCTTCACCTACAGC |
| Human *COL1A1-R* | TCAATCACTGTCTTGCCCCA |
| Human *GAPDH-F* | ATGACCCCTTCATTGACC |
| Human *GAPDH-R* | GAAGATGGTGATGGGATTTC |
| Human *GPX4-F* | ACAAGAACGGCTGCGTGGTGAA |
| Human *GPX4-R* | GCCACACACTTGTGGAGCTAGA |
| Human *LPCAT3-F* | CAGGATACCTGGTCTGCTTCCA |
| Human *LPCAT3-R* | TGAAGAGCCAGTGGATGGTCTG |
| Human *SLC7A11-F* | TCCTGCTTTGGCTCCATGAACG |
| Human *SLC7A11-R* | AGAGGAGTGTGCTTGCGGACAT |
| Human *ACSL4-F* | GCTATCTCCTCAGACACACCGA |
| Human *ACSL4-R* | AGGTGCTCCAACTCTGCCAGTA |
| Mouse *TGF-β1-F* | GCAGTGGCTGAACCAAGGA |
| Mouse *TGF-β1-R* | AGCAGTGAGCGCTGAATCG |
| Mouse *ACTA2-F* | TCCCTGGAGAAGAGCTACGAACT |
| Mouse *ACTA2-R* | AAGCGTTCGTTTCCAATGGT |
| Mouse *COL1A1-F* | AACCCCAAGGAGAAGAAGCA |
| Mouse *COL1A1-R* | AGCGTGCTGTAGGTGAATCG |
| Mouse *GAPDH-F* | TGCCCCCATGTTTGTGATG |
| Mouse *GAPDH-R* | TGTGGTCATGAGCCCTTCC |

**Supplementary Table2.** wt and non-palmitoylated ACAA2 amino sequences

|  | **Amino sequence** |
| --- | --- |
| **wt-ACAA2 sequences** | MALLRGVFIVAAKRTPFGAYGGLLKDFSATDLTEFAARAALSAGKVPPETIDSVIVGNVMQSSSDAAYLARHVGLRVGVPTETGALTLNRL**C**GSGFQSIVSG**C**QEI**C**SKDAEVVL**C**GGTESMSQSPY**C**VRNVRFGTKFGLDLKLEDTLWAGLTDQHVKLPMGMTAENLAAKYNISRED**C**DRYALQSQQRWKAANEAGYFNEEMAPIEVKTKKGKQTMQVDEHARPQTTLEQLQKLPSVFKKDGTVTAGNASGVSDGAGAVIIASEDAVKKHNFTPLARVVGYFVSG**C**DPTIMGIGPVPAINGALKKAGLSLKDMDLIDVNEAFAPQFLSVQKALDLDPSKTNVSGGAIALGHPLGGSGSRITAHLVHELRRRGGKYAVGSA**C**IGGGQGIALIIQNTA |
| **Non-palmitoylated ACAA2 sequences** | MALLRGVFIVAAKRTPFGAYGGLLKDFSATDLTEFAARAALSAGKVPPETIDSVIVGNVMQSSSDAAYLARHVGLRVGVPTETGALTLNRL**A**GSGFQSIVSG**A**QEI**A**SKDAEVVL**A**GGTESMSQSPY**A**VRNVRFGTKFGLDLKLEDTLWAGLTDQHVKLPMGMTAENLAAKYNISRED**A**DRYALQSQQRWKAANEAGYFNEEMAPIEVKTKKGKQTMQVDEHARPQTTLEQLQKLPSVFKKDGTVTAGNASGVSDGAGAVIIASEDAVKKHNFTPLARVVGYFVSG**A**DPTIMGIGPVPAINGALKKAGLSLKDMDLIDVNEAFAPQFLSVQKALDLDPSKTNVSGGAIALGHPLGGSGSRITAHLVHELRRRGGKYAVGSA**A**IGGGQGIALIIQNTA |

### **Key Resources Table**

**3.1Antibodies**

| **Name** | **Supplier** | **Cat no.** |
| --- | --- | --- |
| ACAA2 Antibody | BBI(China) | D121457 |
| Beta-Tubulin Antibody | ZENBIO | 200608 |
| a-SMA Antibody | Proteintech | 14395-1-AP |
| GPX4 Antibody | Proteintech | 67763-1-Ig |
| SLC7A11 Antibody | Proteintech | 26864-1-AP |
| COX4 Antibody | ZENBIO | 350200 |
| GAPDH Antibody | ZENBIO | 380626 |
| Donkey anti-Mouse IgG(H+L) Cross-Adsorbed Secondary  Antibody, Alexa Fluor 488 | Invitrogen | A-21202 |
| Donkey anti-Rabbit IgG(H+L) Cross-Adsorbed Secondary  Antibody, Alexa Fluor 594 | Invitrogen | A-21207 |
| Donkey anti-Mouse IgG(H+L) Cross-Adsorbed Secondary  Antibody, Alexa Fluor 594 | Invitrogen | A-21203 |
| Donkey anti-Rabbit IgG(H+L) Cross-Adsorbed Secondary  Antibody, Alexa Fluor 488 | Invitrogen | A-21206 |

**3.2 Cell lines**

| **Name** | **Supplier** | **Cat no.** |
| --- | --- | --- |
| HepG2 | ATCC | HB-8065 |
| LX2 | Procell | CL-0560 |

**3.3 Organisms**

| **Name** | **Supplier** | **Strain** | **Sex** | **Age** | **Overall n number** |
| --- | --- | --- | --- | --- | --- |
| mice | Cyagen | C57BL/6 | Male | 10 week | 7 |
| mice | Cyagen | ACAA2 KO | Male | 10 week | 7 |
| HFD mice | Cyagen | ACAA2-AAV | Male | 10 week | 7 |
| HFD mice | Cyagen | C57BL/6 | Male | 10 week | 7 |
| CCl_4_ mice | Cyagen | C57BL/6 | Male | 10 week | 7 |
| Human liver | China |  |  |  | 43 |

**3.4 Software**

| **Software name** | **Manufacturer** | **Version** |
| --- | --- | --- |
| Leica LAS Image Analysis | Leica | V3.3 |
| ImageJ | National Institutes of Health | V 1.47 |
| FigDraw |  |  |

**3.5 Other (e.g. drugs, proteins, vectors etc.)**

| **Name** | **Supplier** | **Cat no.** |
| --- | --- | --- |
| Oleic acid (OA) | Sigma-Aldrich | Cat# O1383 |
| Lipo 8000 Transfection Reagent | Beyotime | Cat# C0533 |
| BODIPY 493/503 | Invitrogen | Cat# D3922 |
| Fetal bovine serum | GIBCO | Cat#1009914 |
| DMEM | BI | Cat# 01-052-1A |
| Opti-MEM | GIBCO | Cat# 11058-021 |
| Phosphate Buffered Saline (PBS) | BI | Cat# 02-024-1A |
| DAPI | Beyotime | Cat# C1002 |
| TRIzol reagent | Invitrogen | Cat# 11668019 |
| Protease inhibitor cocktail | Sigma-Aldrich | Cat# P8340 |
| Mito-Tracker Red CMXRos | Beyotime | Cat#C1035 |
| Atglistatin | MCE | Cat#HY-15859 |
| BCA Protein Assay Kit  Triglyceride Assay Kit | Thermo Fisher  Sigma | Cat# 23225  Cat# MAK266 |
| 2-bromopalmitate | Sigma-Aldrich | Cat# 238422 |
| DMEM/F-12 (1:1) medium | VivaCel | Cat#C3130-0500 |
| Mitochondrial Membrane Potential Assay Kit with Rhodamine 123 | Beyotime | Cat#C2008S |
| Palmitic acid (PA) | Sangon biotech | Cat# A423030 |
| RSL3 | Selleck | Cat#S8155 |
| Lip-1 | Selleck | Cat#7699 |
| Compound C (Dorsomorphin) | Selleck | S7840 |
| Lipid Peroxidation MDA Assay Kit | Beyotime | S0131M |
| GSH and GSSG Assay Kit | Beyotime | S0053 |
| Ferrous Ion Content Assay Kit | Solarbio | Cat#BC5415 |
| AICAR | Selleck | Cat#S1802 |
| Nuclear Protein Extraction Kit | Solarbio | Cat#EX1470 |
